# Deciphering the Genomic Traits of Multi-Enterocin-Producing *E. faecium* 1702 from Bottarga: A WGS-Based Characterization

**DOI:** 10.3390/microorganisms14010035

**Published:** 2025-12-23

**Authors:** Abdelkader Fathallah, Mohamed Selim Kamoun, Chaima Hkimi, Kais Ghedira, Mohamed Salah Abbassi, Salah Hammami

**Affiliations:** 1 Tunisian Institute of Veterinary Research, University of Tunis El Manar, Tunis 1006, Tunisia; a.fathallah@yahoo.fr (A.F.); saleehhammami@yahoo.fr (S.H.); 2 Laboratory of Bioinformatics, Biomathematics and Biostatistics (LR16IPT09), Institut Pasteur de Tunis, Tunis 1002, Tunisia; selimkamoun7@gmail.com (M.S.K.); hkimichaima27@gmail.com (C.H.); ghedirakais@gmail.com (K.G.); 3 Higher Institute of Biotechnology of Sidi Thabet, University of Manouba, Ariana BP-66, Manouba 2010, Tunisia; 4 Research Laboratory «Antimicrobial Resistance» LR99ES09, Faculty of Medicine of Tunis, University of Tunis El Manar, Tunis 1006, Tunisia; 5 National School of Veterinary Medicine of Sidi Thabet, University of Manouba, Sidi Thabet, Ariana 2020, Tunisia

**Keywords:** bottarga, *Enterococcus faecium*, bacteriocinogenic, WGS, probiotic/biotechnological/pharmaceutical properties

## Abstract

*Enterococcus* spp. produce diverse bioactive molecules used for biotechnological purposes or as probiotic agents for livestock and human health. The main aim of this study was to decipher the genetic traits using whole-genome sequencing (WGS) of a bacteriocinogenic *Enterococus faecium* 1702 strain showing diverse probiotic traits. Genetic traits of the strain were determined by performing WGS using the NovaSeq6000 platform followed by consecutive sequence analysis using appropriate software. WGS showed that the genome of the *E. faecium* 1702 strain has a size of 2,621,416 bp, with a GC content of 38.03%. The strain belonged to the sequence type ST722 not known as a human clonal lineage. The strain was free of genes encoding clinically relevant antibiotic resistance; in addition, genes encoding *sensu stricto* virulence factors, plasmids, and prophages were not detected. Annotations through the Prokaryotic Genomes Automatic Annotation Pipeline (PGAP) tool revealed 2413 coding sequencing entries (CDC) out of 2521 predicted chromosomal genes. The functional annotation of the whole genome through the KEGG database using KofaScan revealed several genes related to several biological activities, including metabolic process, carbohydrate metabolism, amino acid metabolism, and nucleotide metabolism. The strain harbored three entero-bacteriocins (enterocins) encoded by *entA*, *entB*, and *entX* (enterocin X-alpha and X-beta) genes. Interestingly, the strain harbored the *ansB*, *glsA*, and *arcA* genes encoding L-asparaginase, L-glutaminase, and arginine deiminase, respectively, known for their anticancer activities. *E. faecium* 1702 harbored the *gadB*, *gadC*, and *gadR* genes implicated in gamma(γ)-aminobutyric acid (GABA) production, which is known for its analgesic, anti-anxiety, hypotensive, diuretic, and antidiabetic effects. The WGS findings and phenotypic traits of *E. faecium* 1702 revealed significant features that allow for its use as a probiotic or for biotechnological and pharmaceutical applications.

## 1. Introduction

Lactic acid bacteria (LABs) are Gram-positive bacteria, cocci or rods, non-spore-forming, usually nonmotile, catalase-negative, and fastidious organisms, with a high tolerance for a low pH [1]. LABs belong to the phylum Bacillota (formerly Firmicutes), class Bacilli, and order Lactobacillales, which includes several genera such as *Lactobacillus*, *Lacticaseibacillus*, *Limosilactobacillus*, *Lactiplantibacillus*, *Ligilactobacillus*, *Lactococcus*, *Leuconostoc*, *Pediococcus*, *Streptococcus*, *Aerococcus*, *Alloiococcus*, *Carnobacterium*, *Dolosigranulum*, *Enterococcus*, *Oenococcus*, *Tetragenococcus*, *Vagococcus*, and *Weissella* [2]. LABs are often inhabitants of dairy fermented foods; however, they are also found in fermented vegetables, fermented meat, and fermented cereals [3]. Consequently, LAB strains are used in the food and dairy industry; indeed, they are used as starter cultures for the fermentation of milk, vegetables, meat, fish, cereals, and animal feed in the form of silage as well as in food preservation. Today, they are clearly the most important group of industrial microorganisms, with a market in the range of multi-billion dollars [4]. This is in line with the classification of the vast majority of LAB as ‘Generally Recognized As Safe’ (GRAS) according to the U.S. Food and Drug Administration [5], since they are mainly nonpathogenic, suitable for technological and industrial processes, and acid- and bile-tolerant and can produce antimicrobial substances [6].

Over the last decade, LABs have gained substantial attention for their use as probiotics in both livestock and humans. Probiotics are live microorganisms that confer health benefits when consumed in adequate amounts [7] and numerous studies have highlighted their ability to improve the intestinal balance and overall host function [8,9]. LABs tolerate an acidic pH and bile salts, allowing them to survive passage through the gastrointestinal tract [10], where they can colonize the intestinal mucus, enhance digestion and nutrient absorption, promote growth, and stimulate both specific and non-specific immune responses [11,12]. They also inhibit pathogenic microorganisms by producing antimicrobial metabolites such as lactic acid, hydrogen peroxide, acetaldehyde, diacetyl, and bacteriocins [13]. Beyond their probiotic roles, LABs are widely used in food preservation, contributing to the extended shelf life of fermented foods and the preservation of processed fruits, vegetables, dairy products, and meat [14,15,16].

Numerous investigations, which were conducted to evaluate probiotic LAB candidates and their biotechnological traits, mostly focused on examining their physiological and biochemical traits. However, as high-throughput sequencing technologies have proliferated in recent years, the focus of strain research has shifted to molecular mechanisms and genetic traits [17]. Therefore, the term “probiogenomics” was developed to describe the whole-genome sequencing (WGS) of probiotics as a means of gaining new insights into their functional diversity, metabolic pathways, safety evaluation (virulence factors and antibiotic resistance), and health-stimulating mechanisms [18].

In our previous studies, we reported the occurrence of several bacteriocinogenic *Enterococcus* strains exhibiting probiotics and biotechnological traits, isolated from various sources such as feces of cows, rabbit, poultry, and marine fish intestine [19,20,21,22]. Now, in this study, we investigated LABs from bottarga, which is a culinary specialty of several Mediterranean countries. It is known by names such as “Butarga” in Portugal, Corsica in France, “Bottarga” in Sardinia (Italy), and “Aυγοτάραχο (avgotaracho)” in Greece, and it is produced in Turkey, Egypt, and Tunisia.

Bottarga is a traditional product made from the eggs (fully ripe female roes) of the female mullet (*Mugil cephalus*), blue fin tuna (*Thunnus thynnus*), or yellowfin tuna (*Thunnus albacares*). Bottarga is becoming increasingly popular in the international market and is sold at high prices (more than 200 EUR/kg) [23,24]. Bottarga origins can be traced back to the Middle East 3000 years ago, where Phoenicians were among the first civilizations to cure mullet roe. The first documented bottarga production was in the Nile Delta in the 10th century BC [25]. It was introduced to Tunisia by Jews from Constantinople during Ottoman rule as early as the 16th century [25]. This product is prepared according to old traditional recipes based on salting and drying. It is well known for its organoleptic and nutritional properties. It is a well-fermented food that can be preserved for a long time owing to a very diverse microbiological potential, characterized by the presence of a complex community of microorganisms, notably lactic acid bacteria.

Several fermented foods have been the subject of in-depth studies, such as salted and dried lamb or bauvin meat (kaddid), mesophilic fermented milk (rayeb), etc. However, there are not many studies that have focused on bottarga. It is in this context that we opted for the choice of this substrate in order to recover LAB isolates and to phenotypically evaluate their beneficial properties. In addition, we aimed to decipher the genetic traits using WGS of a multi-enterocin-producing *Enterococcus faecium* strain among 31 recovered bacteriocinogenic LAB isolates. The isolate was selected since it showed a high spectrum of antibacterial activity, susceptibility to clinically relevant antibiotics, tolerance to bile salt, hydrophobicity, and the absence of phenotypic virulence factors.

## 2. Materials and Methods

### 2.1. Isolation of LAB Isolates

Fifty-three samples of bottarga were purchased from a fishmonger who perpetuates ancestral traditions and who is particularly renowned for his expertise and the quality of his product. The occurrence of LAB isolates in samples was investigated according to the protocol previously reported [26] with slight modification. Briefly, from each sample, 25 g was cut into small slices and washed with sterile distilled water to remove dust particles. Then, samples were homogenized in sterile stomachers using 225 mL de Man, Rogosa, and Sharpe (MRS) broth (BD Difco™, Strasbourg, France) and incubated for 4 h at 37 °C. From each sample, 1 mL was serially diluted, plated on MRS agar plates (BD Difco™, Strasbourg, France), and incubated at 37 °C for 24 to 48 h. When growth was observed, from each positive sample, one to ten colonies with a clear zone were randomly selected and then tested by classical bacteriological tests (Gram stain, catalase test, oxidase test). Among collected isolates, only one isolate per sample (Gram-positive cocci, catalase-negative) [26] was kept for further characterization.

### 2.2. Spectrum of Inhibition Against Pathogenic Bacteria

To determine the antibacterial activity, we proceeded as described previously [20,27]. The indicator strains were *Listeria monocytogenes* ATCC 43256, methicillin-resistant *Staphylococcus aureus* [28], *Vibrio alginolyticus* ATCC 17749T [29], *Vibrio anguillarum* ATCC12964T [29], *Escherichia coli* ATCC 25922, *Enterococcus faecalis* JH2-2, vancomycin-resistant *E. faecium* A437 [30], *Salmonella* Typhimurium 4,5,12:i:- (monophasic) LSP 389/97 (kindly provided by María Rosario Rodicio, Departamento de Biología Funcional-Microbiología, Universidad de Oviedo, Oviedo, Spain), *Pseudomonas aeruginosa* ATCC 27853, *Bacillus cereus* (our collection), and *Bacillus subtilis* (our collection). Bacterial cultures were removed from cryogenic storage (Brain Heart Infusion (BHI) broth (Oxoid, Milan, Italy) containing 20% of glycerol, −20 °C) and grown on Mannitol Salt Agar (Microxpress^R^, Accumix, Malaga, Spain) (*S. aureus*), Haecktoen (Titan Biotech Ltd., Delhi, India) (*Salmonella* spp. and *E. coli* strains), Palcam Listeria selective agar (Merck, Darmstadt, Germany) (*L. monosytogenes*), MRS agar (*E. faecalis* and *E. faecium*), Cetrimide agar (*P. aeruginosa*) (Oxoid, Milan, Italy), ChromID Vibrioagar (Biomeriux, Marcy l’Etoile, France) (*Vibrio* spp.), and Brain Heart Infusion (BHI) agar (Oxoid, Milan, Italy) (*Bacilllus* spp.). Then, Petri dishes were incubated overnight at 37 °C. Briefly, the indicator strains were incubated overnight in 5 mL of BHI broth, and then 100 µL (10^8^ UFC/mL) of an overnight culture of the indicator strain was added to 20 mL of BHI broth supplemented with 0.7% agar, mixed, and poured onto a Petri dish. After solidification, the LAB isolates, which were cultured overnight on MRS agar, were placed using a sterile loop on the agar already inoculated with the indicator strains in the form of a streak, with five to seven isolates tested in each Petri dish. Finally, the cultures were incubated overnight at 37 °C [20,27]. The antibacterial activity was visually detected by clear inhibition zones around the tested strain, and scores were assigned based on the diameter of the inhibition halo, as reported previously [20,21,27]. This test was performed in triplicate.

### 2.3. Phenotypic and Molecular Identification of Bacteriocinogenic LAB Isolates

Bacteriocinogenic LAB isolates were initially defined by phenotypic tests, namely, Gram staining, catalase reaction, and species were identified by an Api ^®^20 strep kit (Biomérieux, Marcy l’Etoile, France). For PCR experiments, genomic DNA was extracted as previously reported [21], and the concentrations of DNA samples were measured with NanoDrop (Thermo Scientific, Loughborough, UK). OD_260_/OD_280_ = 1.8–2.0 and OD_260_/OD_230_ = 2.0–2.2 value ranges were considered for PCR. Molecular identification was performed by PCR amplification using the *tuf* gene (specific of *Enterococcus* spp.), as reported by Ke et al. [31], and specific primers for *E. faecalis*, *E. faecium*, *E. gallinarum*, and *E. casseliflavus*/*E. flavescence* species [32] (Table S1).

### 2.4. Antibiotic Susceptibility Testing

The antibiotic susceptibility of all isolates was tested by the disk diffusion method on Mueller–Hinton agar medium (Bio-Rad, Marnes-la-Coquette, France) in accordance with the recommendations of the Clinical and Laboratory Standards Institute guidelines [33]. Isolated strains were initially seeded on MRS plates for 18 to 24 h at 37 °C. Bacterial suspensions were adjusted to obtain an inoculum of approximately 10^8^ CFU/mL (0.5 standard of the McFarland scale) and inoculated onto Müeller–Hinton agar plates with sterile swabs. The following antibiotics were tested (μg/disc): ampicillin (10 μg), erythromycin (15 μg), gentamicin (500 μg), streptomycin (300 μg), linezolid (30 μg), vancomycin (30 μg), teicoplanin (30 μg), and tetracycline (30 μg) (Bio-Rad, Marnes-la-Coquette, France). The plates were incubated at 37 °C for 24 h, and then the diameters of the inhibition zone were measured and the susceptibility of the isolates was interpreted according to the CLSI recommendations [33]. The *E. faecalis* ATCC 25923 reference strain was used as a control.

### 2.5. Gelatinase, Caseinase, DNase Production, and Hemolytic Activity

Gelatinase production was assessed using gelatin agar plates [34]. Petri dishes containing BHI agar (Oxoid, Milan, Italy), supplemented with 30 g l-1 gelatin (Oxoid, Hampshire, England) and 10 g l-1 peptone (Becton-Dickinson Corp., Cockeysville, MD, USA), were streaked with bacteriocinogenic isolates (5–6 streaks per Petri dish) and incubated at 37 °C for 24 h. Then, the plate surface was covered with a saturated solution of ammonium sulfate (Sigma-Aldrich Chemie, Steincheim, Germany). The presence of a transparent zone around the colonies indicates gelatinase activity [35]. Previously characterized enterococcal isolates from our collection [20,21] were used as positive control strains for the above-mentioned tests.

For DNase detection, a colony from an 18 h culture of each tested isolate was streaked onto DNase agar medium (Bio-Rad, Hercules, CA, USA) (5–6 streaks per Petri dish). After incubation for 24 h at 37 °C, plates were flooded with 3% HCl for 5–10 min and we observed whether there was a clear zone around the colonies. *S. aureus* ATCC 25923 was used as a positive control [21].

Hemolytic activity was determined by streaking a single colony on agar plates prepared with Columbia agar (Bio-Rad, Hercules, CA, USA) supplemented with 5% (*v*/*v*) sheep blood. After incubation for 24 h at 37 °C, hemolysis was classified into α-hemolysis (green zone around colonies), β-hemolysis (clear zone around colonies), and γ-hemolysis (no halo around colonies) [36]. *E. faecalis* ATCC 29212 was used as the β-hemolytic strain.

### 2.6. Evaluation of the Probiotic Potential of the Selected Isolates

#### 2.6.1. Growth at Different pH Values

Bacteriocinogenic isolates evaluated as ‘safe’ according to the absence of antimicrobial resistance markers, virulence genes, and phenotypic virulence traits were selected to evaluate the probiotic potential of these isolates. To test the effect of pH on all probiotic candidate strains, the method used was the one described by Lengliz et al. [21] using microtiter plates and MRS broth at pH 4, 5, 6, 7, and 8. The optical density readings of the growth of the bacteria were recorded every hour for 28 hours using a spectrophotometer (Multiscan RC, Labsystems Oy, Helsinki, Finland). Cultures grown in MRS broth served as a control (pH 6.5).

#### 2.6.2. Gastric and Biliary Tolerance

Assessment of resistance to gastric acidity was performed according to the method described by Argyri et al. [37]. Isolates were harvested by centrifugation (10,000× *g*, 10 min, 4 °C), washed twice with sterile phosphate-buffered saline (PBS) (pH 7.3), then resuspended in 1 mL of PBS, and diluted (1:100) in PBS adjusted to pH 2.5. Resistance was assessed in terms of the number of viable colonies and listed in duplicate on Bile Esculin Azid (BEA) agar (BioKar Diagnostics, Allonne, France) after incubation at 37 °C for 0, 0.5, 1, 2, and 3 h, which reflects the time spent by food in the stomach. Bile tolerance was determined by streaking individual colonies on BHI agar plates containing 1% (*w*/*v*) Oxgall bile (BD Biosciences, Oxford, UK). Plates were incubated at 37 °C for 24 h and visually examined for growth.

#### 2.6.3. Hydrophobicity

The hydrophobicity of selected isolates was determined using Congo red staining as reported by Leyva-Madrigal et al. [38]. One colony of each isolate was streaked on MRS agar plates containing 2% NaCl and 0.03% Congo red (Sigma-Aldrich, St. Louis, MO, USA) and incubated at 37 °C for 24–48 h. Red colonies were considered hydrophobic and white or colorless colonies were considered non-hydrophobic [39].

### 2.7. Genome Sequencing, Assembly, and Annotation

We lysed 5–40 microliters of the cell suspension with 120 μL of TE buffer containing lysozyme (final concentration 0.1 mg/mL) and RNase A (ITW Reagents, Barcelona, Spain) (final concentration 0.1 mg/mL), followed by incubation for 25 min at 37 °C. Proteinase K (VWR Chemicals, Radnor, PA, USA) (final concentration 0.1 mg/mL) and SDS (Sigma-Aldrich, St. Louis, MO, USA) (final concentration 0.5% *v*/*v*) were added and incubated for 5 min at 65 °C. Genomic DNA was purified using an equal volume of SPRI beads and resuspended in EB buffer (10 mM Tris-HCl, pH 8.0). Extracted DNA was then quantified with the Quant-iT dsDNA HS kit (ThermoFisher Scientific, Loughborough, UK) assay in an Eppendorf AF2200 plate reader (Eppendorf UK Ltd. Stevenage, UK) and diluted as appropriate. Genomic DNA libraries were prepared using the Nextera XT Library Prep Kit (Illumina, San Diego, CA, USA) following the manufacturer’s protocol with the following modifications: input DNA was increased 2-fold, and the PCR elongation time was increased to 45 s. DNA quantification and library preparation were carried out on a Hamilton Microlab STAR automated liquid handling system (Hamilton Bonaduz AG, Switzerland). Libraries were sequenced on an lllumina NovaSeq 6000 (Illumina, San Diego, CA, USA) using a 250 bp paired-end protocol.

Reads were adapter trimmed using Trimmomatic version 0.30 [40] with a sliding window quality cutoff of Q15. De novo assembly was performed on samples using SPAdes version 3.7 [41], and contigs were annotated using Prokka 1.11 [42].

The *Enterococcus* genome map was predicted using ProkSee (https://proksee.ca/, accessed on 20 July 2024) [43]. PlasmidFinder was used to detect any plasmid [44], and sRNAs within the bacterial genome were identified using the Infernal software [45]. Furthermore, the NCBI Prokaryotic Genomes Automatic Annotation Pipeline (PGAP) (https://www.ncbi.nlm.nih.gov/genome/annotation_prok/ (accessed on 20 July 2024) was used to perform a final annotation. In addition, KofamScan [46], an annotation tool using KOfam, a customized KEGG Orthologs database, was used to annotate the metabolic pathways. Finally, GenoVi, an open-source tool for circular genome visualization, was used for a second map generation as well as the gene distribution related to the cluster of orthologous groups (COGs) classes [47].

### 2.8. The Digital DNA-DNA Hybridization (DDH) Values and Phylogenetic Analyses

The genome sequence data were uploaded to the Type (Strain) Genome Server (TYGS), a free bioinformatics platform available under https://tygs.dsmz.de, for a whole genome-based taxonomic analysis [48]. For the phylogenomic inference, pairwise comparisons between assembled genomes and 17 completely sequenced *Enterococcus*-type strains were conducted using the Genome BLAST Distance Phylogeny approach (GBDP) and accurate intergenomic distances inferred under the algorithm ‘trimming’ and distance formula d5 [49]. DNA–DNA hybridization (DDH) is generally applied when strains share more than 97% 16S rRNA gene sequence identity. DDH values not exceeding 70% are considered an indication that the tested organisms belong to different species [50,51,52]. ANI (Average Nucleotide Identity) is a measure of genomic similarity used to compare the nucleotide sequences of two genomes. Specifically, ANI thresholds are used to classify genomes based on their level of similarity, with 95–96% ANI typically used as a threshold for species-level identification. ANI has been demonstrated to correlate with DDH, where the range of ~95–96% ANI may correspond to the current threshold of 70% DDH similarity [53]. DDH values and confidence intervals were calculated using the recommended settings of the GGDC 4.0 [54,55]. The type-based species clustering using a 70% dDDH radius around each of the 17 type strains was performed as previously described [48]. Subspecies clustering was carried out using a 79% dDDH threshold as previously introduced [49]. The phylogenetic tree constructed using the TYGS server (https://tygs.dsmz.de) based on complete genomes was visualized using iTol v.6.8.1.

### 2.9. Comparative Genomics

Genome comparison of the *E. faecium* strain 1702 genome with related species was performed using BRIG (Blast Ring Image Generator), an open-source multi-platform software application, which displays multi-genome comparisons and similarity of the reference genome at the center of one image compared to other multiresistant strains, in the form of a concentric colored ring set according to BLASTn identity [56]. Furthermore, the *E. faecium* 1702 proteome revealed through PGAP was compared to proteome sequences of *E. faecium*-related species. Orthologous genes and the shared gene core were also determined using the Reciprocal Best Hits (RBH) Basic Local Alignment Search Tool (BLASTP) with an expectation (E) value threshold of 10^−5^ [57].

### 2.10. Virulence and Antibiotic Resistance Genes’ Identification

The investigation of antimicrobial resistance (AMR) genes was conducted through two tools for a more comprehensive approach. Staramr [58] was used, which is a tool that compares the bacterial assemblies against known databases such as Resfinder and Plasmidfinder, often used to predict drug resistance phenotypes in the case of microbiological resistance, alongside the Resistance Gene Identifier (RGI) using the Comprehensive Antibiotic Resistance Database (CARD) [59]. Virulence factors were later screened through the virulence factor database (VFDB) (http://www.mgc.ac.cn/VFs/ accessed on 25 July 2024) [60].

### 2.11. Carbohydrates’ Utilization and Related Genes

Carbohydrate metabolism-related genes were identified through the integration of KofamScan and KEGG for metabolic pathways’ identification. The carbohydrate-active enzymes (CAZymes) were investigated using the dbCAN3 server [61] and based on the latest version of the Carbohydrate Active Enzymes Database (CAZy) [62] with a coverage > 0.35 and an E-value cut-off < 10^−15^.

### 2.12. Bacteriocin Genes’ Identification

Bacteriocin-related genes were investigated and identified using Bagel4 [63], a bacteriocin genome mining tool that enables the identification as well as the annotation of bacteriocin gene clusters. The results were then compared to the Bactibase database (BACTIBASE web server is freely accessible at bactibase.pfba-lab.org) [64].

### 2.13. Mobile Genetic Elements

PHASTEST (PHAge Search Tool with Enhanced Sequence Translation) was used to check for a possible prophage presence within *E. faecium* strain 1702 [65]. The MGEfinder(v1.0.6) tool was also used to assess the presence of mobile genetic elements other than genes related to antimicrobial resistance and virulence [66].

### 2.14. Complete Genome Sequence Data Accession Number

The sequence data for the *Enterococcus faecium* strain 1702 genome was deposited in GenBank under the accession number JBFCWA000000000.

## 3. Results

### 3.1. Selection of Bacteriocinogenic LAB Isolates

During this study, 53 LAB isolates were collected. Among them, 31 isolates showed inhibition of at least one indicator strain (Table S2). Indeed, eight isolates inhibited *L. monocytogenes*, and each of the *E. faecalis* and *E. faecium* isolates were inhibited by seven isolates, whereas the MRSA isolate was inhibited by only two isolates. Concerning *Vibrio* species, nine and three isolates inhibited the growth of *V. anguillarum* and *V. alginolyticus*, respectively. Finally, *E. coli* ATCC 25922, *A. baumannii* 8327, *P. aeruginosa* 46805, and *S.* Typhimurium LSP 389/97 were inhibited by five, five, two, and three isolates, respectively. None of the isolates was able to inhibit *B. cereus*. The inhibition spectrum of the 32 isolates was conserved by treating the neutralized supernatants with proteinase K, indicating the proteic nature of the inhibitor substances.

### 3.2. Identification of LAB Isolates

Through PCR experiments and Api 20 Strep, among the 31 LAB isolates, 28 were identified as *Enterococcus* spp. and belonged to the following species: *E. faecalis* (*n* = 16), *E. faecium* (*n* = 10), *E. casseliflavus* (*n* = 3). The three non-enterococci isolates were identified by Api 20 Strep as *Lactococcus lactis* subp. *Lactis* (Table S2).

### 3.3. Detection of Genes Encoding Bacteriocins

When using PCR experiments, among the nine enterocin genes investigated, eight isolates showed the *entB* gene and seven contained the *ent1071A*/B gene. Interestingly, the *E. faecalis* P42 isolate co-harbored *entB* and *ent1071A*/B genes (Table S1) and one isolate *E. faecium* S7 harbored *entA*, *entB,* and *entX* genes. The remaining genes were not detected.

### 3.4. Safety Assessment of the 31 Bacteriocinogenic Isolates

Antimicrobial susceptibility showed that the majority of bacteriocinogenic isolates were susceptible to the tested antibiotics, except three isolates showing resistance to some antibiotics. *E. faecium* S7 (after called *E. faecium* 1702) was resistant to rifampicin, ciprofloxacin, trimethoprim, and trimethoprim-sulfamethoxazole. *E. faecium* S19 was phenotypically resistant to linezolid, ampicillin, erythromycin, and tetracycline, whereas *E. faecalis* S44 was resistant to vancomycin (MIC > 256 µg/mL), linezolid (MIC = 96 µg/mL), ampicillin, erythromycin, and tetracycline. Fifteen isolates were gelatinase producers; however, only one isolate was able to degrade DNA (Table S2) and none were beta-hemolytic.

Among the isolates that displayed probiotic traits and lacked virulence genes as well as acquired antimicrobial resistance markers, the *E. faecium* S7 strain (hereafter referred to as *E. faecium* 1702) was selected for whole-genome sequencing. This strain was chosen based on its broad inhibitory spectrum, antibiotic susceptibility profile (absence of clinically relevant resistance), lack of key virulence factors (hemolysis and gelatinase), tolerance to bile salts, ability to grow at pH 4, and cell-surface hydrophobicity, to evaluate its potential for use as a probiotic candidate or as a starter.

### 3.5. General Characteristics of the E. faecium 1702 Strain Genome

The genome of *E. faecium* 1702 comprises 2,621,416 bp, with a GC content of 38.03%, an N50 equal to 531,479, a total number of contigs of 35, and a number of ambiguous characters N equal to null, with the largest contig size equal to 840,477. The strain belongs to sequence type 722 (ST722). The genomic characteristics of this strain are summarized in Table 1 and Figure S1. Annotations through the PGAP tool revealed 2413 coding sequencing (CDS) out of 2521 predicted chromosomal genes alongside three kinds of non-coding RNAs, including 16S rRNA (*n* = 8), 5S rRNA (*n* = 4), 23S rRNA (*n* = 4), tRNA (*n* = 60), and sRNA (*n* = 19). No plasmids, no phages, and no clustered, regularly interspaced short palindromic repeats (CRISPR) consensus were detected. A visual representation of the draft DNA genome sequence of *E. faecium* strain 1702 is included in Figure S1.

### 3.6. Phylogenetic Analysis

The phylogenetic tree generated based on a whole-genome-based taxonomic analysis through the Type (Strain) Genome Server (TYGS) revealed four species and subspecies clusters. The strain 1702 was assigned to the fourth cluster, corresponding to *E. faecium* species including *E. faecium* strain 2001564, *E. faecium* strain WEswe-R, *E. faecium* strain T17-1, *E. faecium* strain ef332, *E. faecium* strain AT02, *E. faecium* strain ww sludge entV1, *E. faecium* strain AT44, *E. faecium* strain AT45b, and *E. faecium* strain H7.

**Figure 1:**
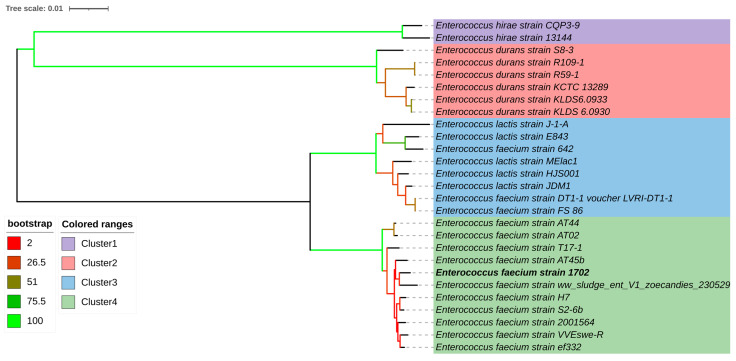
Phylogenetic tree of *Enterococcus faecium* strain 1702 and related species based on 16S rRNA sequences.

**Figure 2:**
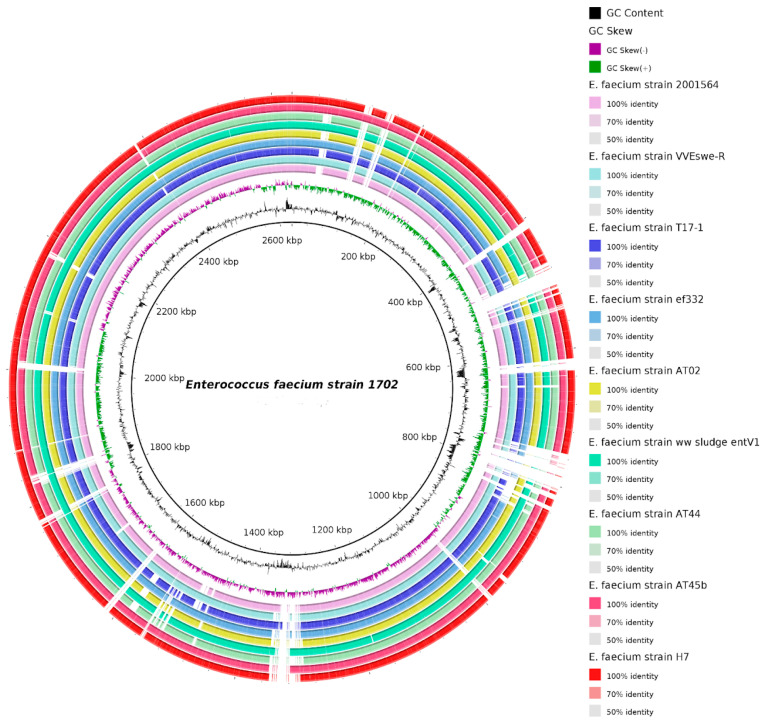
Multiple genome alignment of *Enterococcus faecium* strain 1702 with related strains with the BRIG. Different colors represent different whole-genome strains. Nine genomes were compared against the *E. faecium* strain 1702. The strains used for the comparison were *E. faecium* strain 2001564, *E. faecium* strain WEswe-R, *E. faecium* strain T17-1, *E. faecium* strain ef332, *E. faecium* strain AT02, *E. faecium* strain ww sludge entV1, *E. faecium* strain AT44, *E. faecium* strain AT45b, and *E. faecium* strain H7.

ANI (Average Nucleotide Identity), a measure of genomic similarity, was used to compare the nucleotide sequences of two genomes. Values surpassing 97% are indicative of the same species while those falling below 95% indicate different species. The results demonstrate that closely related *E. faecium* species strains have ANI values exceeding 99%, with *E. faecium* strain 1702 exhibiting the highest similarity to *Enterococcus faecium* strain H7 (CP083179.1) presenting an ANI value of 99.41, followed by *Enterococcus faecium* strain S2-6b (CP088191.1) with an ANI value of 99.34, *Enterococcus faecium* strain ww_sludge_ent_V1_zoecandies_230529 (CP165618.1) with a value of 99.25, *Enterococcus faecium* strain ef332 (CP058891.1) with a value of 99.23, *Enterococcus faecium* strain AT45b (CP097022.1) with an ANI value equal to 99.22, *Enterococcus faecium* strain 2001564 (CP086643.1) with an ANI equal to 99.19, and *Enterococcus faecium* strain VVEswe-R (CP041261.3) and *Enterococcus faecium* strain T17-1 (CP109839.1) with ANI values of 99.03 and 99.02, respectively (Figure 1 and Figure 2). *E. faecium* strain sludge entV1 (NZ_CP165618.1) was isolated from sewage sludge in China (Hangzhou, Zhejiang) (https://www.ncbi.nlm.nih.gov/nuccore/CP165618.1/ accessed on 18 Augustus 2025); meanwhile, *E. faecium* strain AT45b (NZ_CP097022.1) (https://www.ncbi.nlm.nih.gov/nuccore/CP097022.1 accessed on 18 Augustus 2025) is a florfenicol and linezolid-resistant (harboring the *poxtA* gene) strain isolated from a raw poultry meat-based diet for companion animals imported from Germany to Switzerland [67].

### 3.7. Genotype Analysis for Carbohydrate Utilization in E. faecium Strain 1702

**Figure 3:**
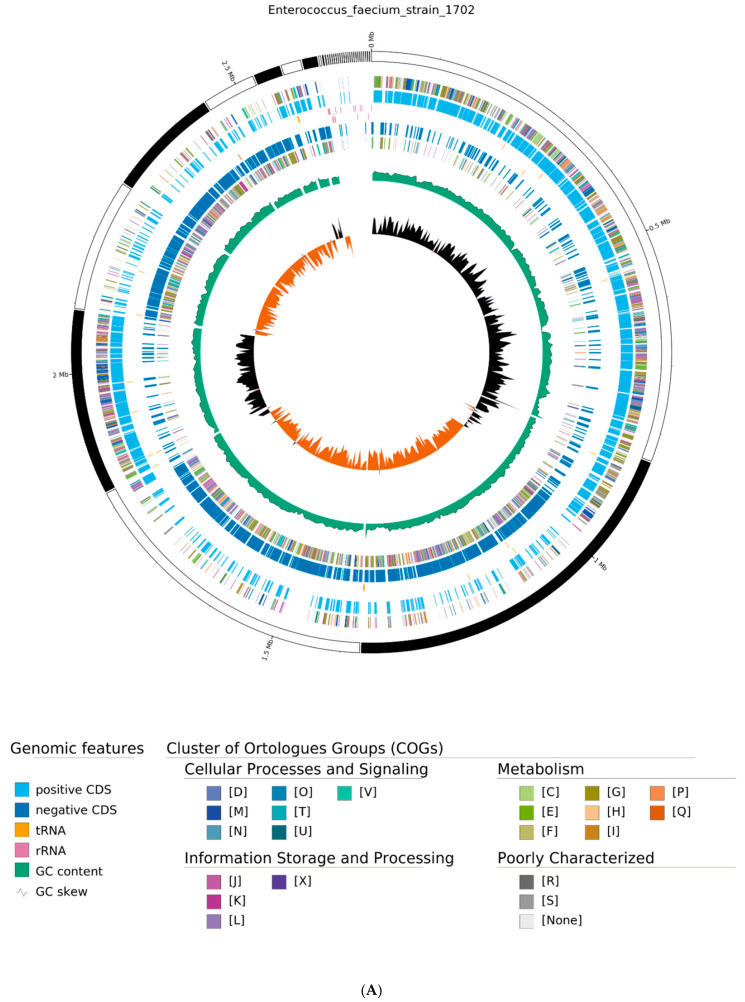

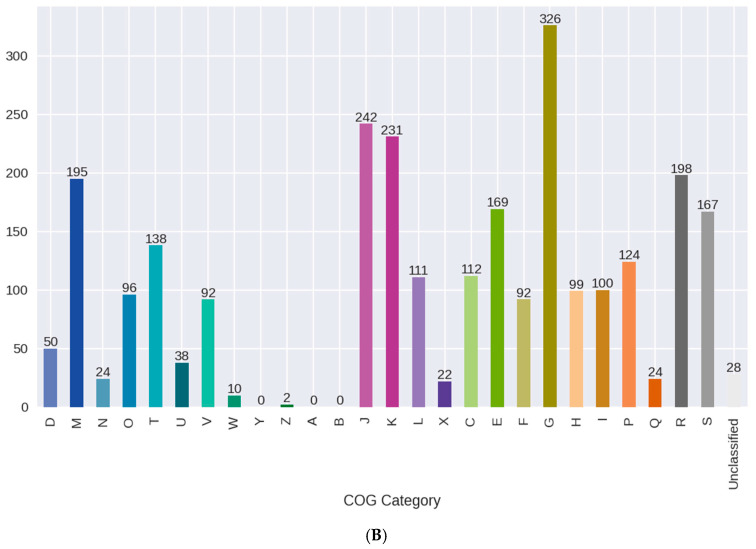
*Enterococcus faecium* strain 1702 genome map and COG distribution. (**A**) Maps of *E. faecium* strain 1702. The circular illustration was visualized using the GenoVi server. In the cycle diagram, the first and fourth circles from the outside represent CDSs on the positive and negative strands, respectively, with different colors denoting different COG function classifications. The second and third circles represent CDSs, tRNA, and rRNA on the positive and negative strands, respectively. The fifth circle indicates the GC content, and the sixth circle represents the GC-Skew value. (**B**) The gene distribution related to the COG classes. A: RNA processing and modification; B: chromatin structure and dynamics; C: energy production and conversion; D: cell cycle control and mitosis; E: amino acid metabolism and transport; F: nucleotide metabolism and transport; G: carbohydrate metabolism and transport; H: coenzyme metabolism; I: lipid metabolism; J: translation; K: transcription; L: replication and repair; M: cell wall/membrane/envelope biogenesis; N: cell motility; O: post-translational modification, protein turnover, chaperone functions; P: inorganic ion transport and metabolism; Q: secondary structure; T: signal transduction; U: intracellular trafficking and secretion; Y: nuclear structure; Z: cytoskeleton; R: general functional prediction only; S: function unknown, V: defense mechanism.

The RAST analysis identified 273 subsystems in the genome of *E. faecium* 1702. The most abundant COGs are carbohydrate metabolism and transport (G: 326), translation (J: 242), transcription (K: 231), general functional prediction only (R: 198), cell wall/membrane/envelope biogenesis (M: 195), amino acid metabolism and transport (E: 169), function unknown (S: 167), signal transduction (T: 138), inorganic ion transport and metabolism (P: 124), energy production and conversion (C: 112), replication and repair (L: 111), lipid metabolism (I: 100), coenzyme metabolism (H: 99), post-translational modification, protein turnover, chaperone functions (O: 96), nucleotide metabolism and transport (F: 92), and the defense mechanism (V: 92) (Figure 3).

### 3.8. Genomic Analysis of Carbohydrate Utilization and Related Genes

**Figure 4:**
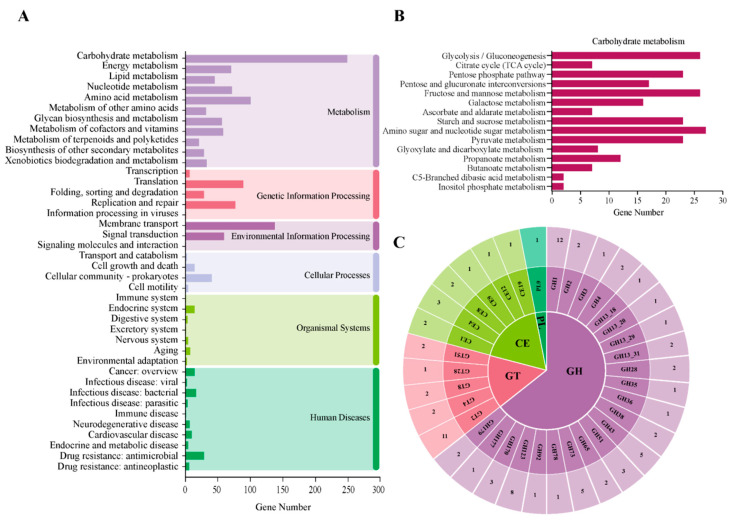
Genotype analysis for carbohydrate utilization in *E. faecium* 1702. (**A**): Kyoto Encyclopedia of Genes and Genomes (KEGG) functional annotation. The functional categories are represented as follows: Light purple: metabolism; Light Red: genetic information processing; Blue gray: cellular processes; Purple: environmental information processing; Yellow–green: organismal systems, Green: human diseases. (**B**): Distribution of CAZymes. Different colors represent different classes of CAZymes identified in the genome; GH: glycoside hydrolase, GT: glycosyl transferase, CE: carbohydrates esterases, PL: polysaccharide lyases. Represented from the inner to the outer rings are CAZyme classes, CAZyme families, and the number of genes identified in each family, respectively. (**C**): Carbohydrate metabolism.

The functional annotation of the *E. faecium* strain 1702 whole genome through the KEGG database using KofamScan revealed several genes and pathways belonging to several major types of biological processes (Figure 4). Among them, the pathways related to metabolic processes presented an important number of involved genes. Carbohydrate metabolism displayed one of the largest gene abundances, with 226 genes in several pathways (Figure 4A,B). Amino acid metabolism and nucleotide metabolism followed, with 91 and 65 genes, respectively (Figure 4A).

The CAZyme search based on the CAZY database data revealed four CAZyme families (Figure 4C): GHs (glycoside hydrolase) (58 enzymes belonging to 22 families), which are involved in the formation of glycosidic bonds, GT (glycosyltransferase) (18 enzymes belonging to 5 families) modulating the hydrolysis and rearrangement of glycosidic bonds along with a few CEs (carbohydrate esterases) (10 enzymes belonging to 6 families), and PL (one polysaccharide lyase, a thiopeptidoglycan lyase (EC 4.2.2.-) belonging to the PL9 family). A total of 88 genes were detected across 35 sub-families (Figure 4C). GH2, GH13, and GH32 have been reported as major oligosaccharide-degrading enzymes. In our strain, GH2 and GH13 were presented by two and five enzymes, respectively; however, GH32 was not detected.

The largest number of carbohydrate genes is part of amino sugar and nucleotide sugar metabolism (27 genes), closely followed by glycolysis/gluconeogenesis and fructose mannose metabolism with 26 genes for each, while inositol phosphate metabolism and C5-branched dibasic acid metabolism are the least abundant, with two genes for both metabolic pathways (Figure 4B).

### 3.9. DNA Biosynthesis

The production of enzymes implicated in DNA biosynthesis is indicative of folic acid biosynthesis, which is required for nucleic acid synthesis and crucial for the growth of the fetal nervous system in the host. Enzymes involved in the guanine nucleoside triphosphate (GTP) pathway have been identified, namely, dihydroneopterin aldolase [*folA* (EC1.5.1.3) and *folB* (EC1.13.11.81, EC4.1.2.25, EC5.1.99.8)], folypolyglutamate synthase [*folC* (EC6.3.2.12, EC6.3.2.17)], methylenetetrahydrofolate dehydrogenase [*folD* (EC1.5.1.5, EC3.5.4.9)], dihydropteroate synthase (DHPS) [*folP* (EC2.5.1.15)], and guanosine triphosphate (GTP) cyclohydrolases [*folE* (EC2.7.6.3, EC3.5.4.16)].

### 3.10. Determination of Probiotic Attributes

#### 3.10.1. Occurrence of Genes Encoding Bacteriocins 

**Figure 5:**
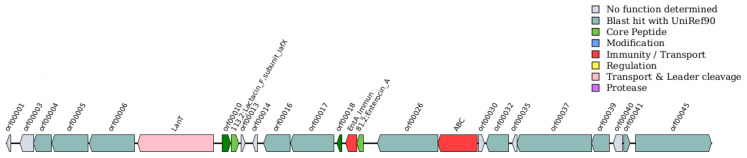
Bacteriocin gene cluster of enterocin A (see Table S3 for further detail of gene content and functions).

**Figure 6:**
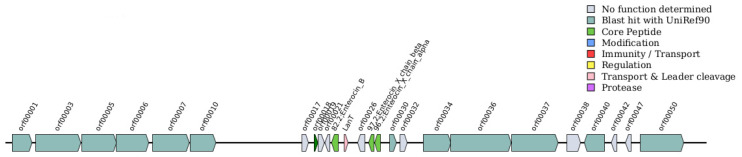
Bacteriocin gene cluster of enterocin B and enterocin X (see Table S4 for further detail of gene content and function).

The bacteriocin screening of *E. faecium* 1702 using BAGEL4 identified three major enterocins: enterocin A (*entA*), enterocin B (*entB*), and the two-component enterocin X (Xα and Xβ) encoded by *entX*. Notably, the genes encoding enterocin B and enterocin X (Xα and Xβ) are located within the same genomic cluster, with their structural genes positioned in close proximity. The complete gene clusters for the three enterocins are shown in Figure 5 and Figure 6, while detailed compositions of the two clusters are provided in Supplementary Tables S3 and S4.

#### 3.10.2. Gut Colonization and Adaptation

Genome annotation was examined for the presence of probiotic-associated genes to confirm the probiotic potential of *E. faecium* 1702. This analysis revealed multiple genes involved in adhesion, the stress response, temperature tolerance, and bile resistance. Notably, the strain harbored several adhesion-related genes, including *fbpA* (fibronectin-binding protein A), *eno* (enolase), *lspA* (lipoprotein signal peptidase II), *tuf* (elongation factor Tu), and the LPXTG-motif cell wall-anchored protein (*srtA* sortase), all of which support a strong adhesion capacity. In addition, five genes—*celA*, *celB*, *celC*, *celD*, and *celR*, encoding components of the phosphoenolpyruvate-dependent sugar phosphotransferase system (PTS) associated with gut persistence—were identified (Table S5).

*E. faecium* 1702 also contained genes related to gastrointestinal survival, encoding proteins that are crucial for maintaining the structural integrity of the bacteria, enabling them to withstand conditions similar to those observed in the gastrointestinal tract, such as bile tolerance. In the *E. faecium* 1702 strain, bile tolerance is encoded by several genes, including *ppaC* (manganese-dependent inorganic pyrophosphatase (EC3.6.1.1); maintains surface tension and keeps membrane integrity), *cfa* (cyclopropane-fatty-acyl-phospholipid synthase (EC3.6.1.1); enhances lipid synthesis), *glnA* (UDP-galactopyranose mutase (*glf*) glutamine synthetase (EC 6.3.1.2)), *arsB* (sodium bile acid symporter (ko:K03325)), *mleS* (malolactic enzyme (MLE) (EC1.1.1.38, EC4.1.1.101)), *murE* (murE synthease (EC6.3.2.13, EC6.3.2.7)), *murF* (murF synthetase (EC6.3.2.10)), sodium proton antiport genes (*nhaK* (ko:K03316)*, napA*), cholylglycine hydrolase (*cbh*), and oligopeptide transporting proteins (*oppA*, *oppB*, *oppC*, *oppD*, *oppF*) (Supplementary File, Table S5). In addition, several genes encoding cold and heat stress were identified, including chaperones (*dnaK*, *dnaJ*, *hslO*), cold shock proteins (*cspA*, *cspB*, *cspC*, *cspD*), and heat shock proteins (*grpE*, *groES*, *groEL*, *ctsR*, *hslV*, *hslU*).

Resistance to gastrointestinal pH-induced stress is extremely crucial for the probiotic potential. The strain contained eight genes encoding ATP synthase subunits as well as *nhaA*, *nhaC*, and *nhaP1* genes encoding Na (+)/H (+) antiporter NhaA, NhaC, and NhaP1, respectively. Meanwhile, three potential genes (*asp1*, *aspS*, WQ51_04310, and *yloU*) encoding alkaline shock proteins existed in the strain *E. faecium* 1702 (Supplementary File, Table S5). In addition, the genome contained two multi-component transport ATP-binding protein-encoding genes, *opuAA*, *opuCA*, *opuCB*, *opuCC*, *opuCD*, and *proWX*, which are responsible for the uptake and accumulation of osmoprotectants such as glycine betaine or proline, adapting to the persistent gastrointestinal osmotic stress stimulation. Additionally, the whole genome contained a complete NADH system (NADH peroxidase and NADPH reductase) (*npr* and *nox* genes), thioredoxin reductase (*trxB*) and thioredoxin (*ytpP* and *trxA* genes), and glutaredoxin (*nrdH*), implicated in oxidative stress. Also, the strain harbored the *sodA* gene (EC1.15.1.1) encoding superoxide dismutase, which destroys radicals normally produced within the cells and which are toxic to biological systems. Other genes encoding proteins related to the response to oxidative stress were also observed, such as manganese transport system permease proteins (encoded by *mntA*, *mntB*, and *mntC* genes), acting as a functional replacement for superoxide dismutase. Also, the strain harbors the methionine sulfoxide reductase system (*msrA* and *msrB* genes), which can catalyze the oxidized methionine residues formed by ROS and RNS in proteins (Supplementary File, Table S5). Also, the strain harbored the *sodA* gene encoding superoxide dismutase (EC 1.15.1.1).

Additionally, the *E. faecium* 1702 genome carries several genes associated with immunomodulatory functions, including the *dltA–dltD* genes of the *dlt* operon, as well as *rpoB* (EC 2.7.7.6), *fusA* (ko:K02355), and *pyrG* (EC 6.3.4.2) (Table S5).

#### 3.10.3. Vitamin and Bioactive Compounds’ Production

Several genes implicated in the metabolism of terpenoides and polyketides (20 genes), xenobiotic biodegradation and metabolism (35 genes), and metabolism of the cofactor and vitamins (55 genes) were also detected (Figure 4A).

Concerning vitamin production, genome annotation of *E. faecium* 1702 showed the occurrence of several genes implicated in the metabolism of the cofactor and vitamins (55 genes) (Figure 4A). These vitamins include vitamin B7 (Biotin) [*birA* (EC 6.3.4.15. biotin-protein ligase), bioY (substrate-specific component BioY of biotin ECF transporter)]; B1 (thiamine) [(*thiN* (EC 2.7.6.2; thiamin pyrophosphokinase, vitamin B1-binding domain), *pfkB* (EC 2.7.1.56), *tenA* (thiaminase II, EC3.5.99.2), *thiE* (thiamin-phosphate pyrophosphorylase, EC 2.5.1.3), *thiM* (hydroxyethylthiazole kinase, EC 2.7.1.50)], B2 (riboflavin) [(*ribH* (EC2.5.1.78), *ribBA* (EC 3.5.4.25, EC4.1.99.12), *ribE* (EC2.5.1.9), *ribF* (EC2.7.1.26, EC 2.7.7.2), *ribD* (EC1.1.1.193), *ribU* (riboflavin ECF transporter)]; and B6 (pyridoxin) [(*thiD* (EC 2.7.1.35), *serD* (EC 2.6.1.52), *gap* (EC 1.2.1.12), *dxs* (EC 2.2.1.7)], menaquinone (K2), and phylloquinone (K) biosynthesis [(*menB* (EC4.1.3.36), *menG* (EC2.1.1.163), *menE* (EC6.2.1.26), *menC* (EC4.2.1.113)]. In addition to their role in DNA biosynthesis, folic acid belongs to the B-group vitamins, and as mentioned above in section ‘3.9. DNA biosynthesis’, genes (*folA, folB*, *folC, folD*, *folE, folP*) implicated in folic acid were detected in the *E. faecium* 1702 strain.

Gamma(γ)-aminobutyric acid (GABA) is a four-carbon non-protein amino acid. It has its amino group on the γ- instead of the α-carbon and a relative molecular weight of 103.1 kDa. GABA is widely distributed in nature among microorganisms, plants, and animals. *E. faecium* 1702 harbored the *gadB* (EC4.1.1.15; EC4.1.2.27) gene encoding glutamate decarboxylase beta (GAD) and the *gadC* (glutamate/gamma-aminobutyrate antiporter) (ko:K20265) gene, which is responsible for importing L-glutamine and exporting either glutamate or GABA. Both of these genes form a *gad* operon, which is positively regulated by *gadR,* encoding the GadR protein. Therefore, *gad* genes have the potential to aid strains in producing the maximum GABA concentration under harsh conditions, and they are also linked to probiotic properties.

Interestingly, the genome annotation showed the occurrence of a phytase (inositol-phosphate phosphatase) enzyme encoded by the *suhB* gene (EC3.1.3.25; ko:K01092) previously identified in *E. coli* [68].

#### 3.10.4. Genes Possibly Involved in Heavy Metals’ Removal in the Genome of *E. faecium* 1702

Genome annotation of the strain showed several genes implicated in heavy metal uptake and tolerance, including *mntA* (manganese transport system ATP-binding protein), *mntB*/*mntC*/*mntH* (manganese transport system permease proteins), *czrA* (metalloregulator ArsR/SmtB family transcription factor (arsenical resistance operon repressor), ko:K22043), *copA* (EC3.6.3.54 *copZ* (heavy-metal-associated domain, ko:K07213), *zurR* (Ko:K02076) (zur; Fur family transcriptional regulator, zinc uptake regulator), XK27_05385 (ZIP zinc transporter, ko:K07238), *psa4* (zinc transporter, ko:K09815), *czcD* (cobalt–zinc–cadmium efflux system protein, ko:K16264), *spx4* (arsenate reductase (glutaredoxin) activity (a metal sequestering protein), EC1.20.4.1), *cadA* (P-type ATPase (extrusion of Cu^+^ and Ag^+^), EC3.6.3.3, EC3.6.3.5), *copA* (a P-type ATPase dedicated to copper efflux, EC3.6.3.54), and *copB* (a P-type ATPase dedicated to copper extrusion, EC3.6.3.4).

#### 3.10.5. Safety Assessment of *E. faecium* 1702

##### Antibiotic Resistance Genes’ Identification

The *E. faecium* 1702 strain harbored two copies of the *vanY* gene of the *vanB* cluster encoding glycopeptide (vancomycin and teicoplanin) resistance, the *aac*(6′)-Ii encoding the chromosomally encoded enzyme sminoglycoside N-acetyltransferase AAC(6′)-Ii (AB1A69_12035), and the *efmA* gene (AB1A69_00400) encoding the multidrug efflux MFS transporter EfmA responsible for macrolide and fluoroquinolone resistance through antibiotic efflux mechanisms. The AMR gene search also revealed the presence of the innate *msrC* gene (AB1A69_11335; AB1A69_05860) encoding the ABC-F-type ribosomal protection protein Msr(C) related to macrolide, lincosamide, and streptogramin resistance as well as the presence of the *efrA* (AB1A69_08915) gene coding the multidrug efflux ABC transporter subunit EfrA, leading to resistance toward macrolide antibiotics, fluoroquinolone, and rifamycin antibiotics. In addition to these genes, we also noted the presence of eatAv (AB1A69_00840) coding for ABC-F-type ribosomal protection-like protein Eat(A) responsible for pleuromutilin antibiotic resistance. Finally, it harbors *fosI,* encoding fosfomycin resistance (Table 2).

##### Genes Encoding Virulence Factors

*E. faecium* strain 1702 harbored 21 genes (Table 3) involved in different cellular functions and corresponding to (1) the encoding cell wall-anchored proteins involved in surface adhesion to the extracellular matrix (*acm*-Entls, *scm*-Entfm-hosp, *fnm*-Entfm-com, *sgrA*-Entfm-hosp, *fms11*-Entfm-hosp, *fms13*-Entfm-hosp, *fms14*-Entfm-hosp, *fms15*-Entfm-hosp, *fms16*-Entfm-hosp, *fms17*-Entfm-hosp, *fms19*-Entfm-hosp, *empA*-Entfm-hosp, *empB*-Entfm-hosp, *empC*-Entfm-hosp), (2) a role in carbohydrate metabolism and cell growth (*ccpA*-Entfm-com), (3) biofilm formation (*sag*-Entfm-hosp), (4) encoding general stress proteins important in bile salt tolerance and general intestinal adaptation (*gls20*-Entfm, *gls33*-Entfm, *glsB*-Entfm, *glsB1*-Entfm), and (5) encoding phosphotransferase systems important in carbohydrate transport (*bepA*-Entfm-com) (Table 3). Interestingly, the genes encoding the most important virulence factors were not detected, namely *hylA*/*hylB* (encoding hyaluronidase), *gelE* (gelatinase), *esp* (enterococcal surface protein), *hla*, *hlb*, *hld*, and *hlg* (all encoding hemolysin).

##### Biogenic Amines’ Synthesis

Given the potentially harmful neuroactive properties of biogenic amines (BAs) [69], their concentrations in food products should be strictly regulated. Genes related to the formation of biogenic amines, i.e., lysine decarboxylase (EC4.1.1.18), ornithine/lysine decarboxylase (EC4.1.1.116), arginine decarboxylase (EC4.1.1.19), agmatinase (EC3.5.3.11), spermidine synthase (EC2.5.1.16), arginase (EC3.5.3.1), ornithine decarboxylase (EC4.1.1.17), histidine decarboxylase (EC4.1.1.22), tyrosine decarboxylase (EC4.1.1.25), and tryptophan decarboxylase (EC4.1.1.28), were not found in the E. faecium strain 1702 genome. Therefore, regarding the biosynthesis of biogenic amines, the use of the E. faecium strain 1702 in food products meets the requirements of food safety.

##### Mobile Genetic Elements

As mentioned above, the strain was free of plasmids. Prophage analysis of the *E. faecium* 1702 genome did not reveal any significant hits, indicating the absence of detectable prophage-related genes. In contrast, mobile genetic element (MGE) screening identified the IS*Efa10* (*AB1A69_00090*) gene, encoding an IS*3* family transposase, as well as the *TnpB* (*AB1A69_11995*) gene, coding for an RNA-guided endonuclease of the TnpB family.

### 3.11. Other Beneficial Traits

#### 3.11.1. Anticancer Genes and Cholesterol-Lowering Potential

Several enzymes were confirmed to present anticancer activities, including L-asparaginase, L-glutaminase, L-arginase (Arginine deiminase), and L-methioninase [70]. Therefore, genome annotation was screened for the presence of genes encoding these enzymes. The strain contained the *ansB* (Ec3.5.1.1), *glsA* (EC3.5.1.2), and *arcA* (EC 3.5.3.6) genes encoding L-asparaginase (Asparagines amidohydrolase), L-glutaminase, and arginine deiminase, respectively.

Interestingly, the strain harbors cholylglycine hydrolase (EC 3.5.1.24), also known as bile salt hydrolase (BSH), encoded by the *cbh (bsh)* gene, which plays a key role in bile salt tolerance. This enzyme catalyzes the hydrolysis of glycine- and taurine-conjugated bile salts into amino acid residues and free bile acids. Consequently, bile salt deconjugation by BSH can contribute to lowering serum cholesterol levels [71].

#### 3.11.2. Genes that Contribute to Organoleptic Properties

The strain harbored the *mccB* and *metB* genes. The *mccB* gene encodes a cystathionine gamma-lyase (EC4.4.1.1; EC4.4.1.2; EC4.4.1.8) (also known as cystathioninase or gamma-cystathionase), whereas the *metB* gene encodes cystathionine beta-synthase (EC2.5.1.48) (also known as cystathionine synthetase, cystathionine synthase, or cystathionine beta-lyase (CBL)). The enzymatic degradation of amino acids in cheese is believed to generate aroma compounds and therefore to be essential for flavor development. Cystathionine beta-lyase (CBL) can convert cystathionine to homocysteine but is also able to catalyze an alpha, gamma elimination. With methionine as a substrate, it produces volatile sulfur compounds, which are important for flavor formation in Gouda cheese.

Genome analysis showed the presence of several genes encoding enzymes potentially related to the aromatic profile of the wine, mainly harbored by strains belonging to the *Oenococcus* genus [72], including genes encoding the phenolic acid carboxylase (*padC*; ko:K13727), carbamate kinase (*arcC*; EC 2.7.2.2, ko:K00926), and arginine deiminase (*arcA*; EC3.5.3.6). Interestingly, *arcA* and *arcC* are clustered with other genes (*arcD*, *arcT*, *argS*, *argR*), forming the arginine deiminase pathway gene cluster, as reported in *Lactococcus Lactis* [73]. In addition, the *bglC* (EC3.2.1.21, EC3.2.1.85, EC3.2.1.86), *blgC1* (EC3.2.1.21, EC3.2.1.52), and *bglH1* (EC3.2.1.21, EC3.2.1.86) genes encoding beta-glucosidase enzymes as well as the *abfA* (EC3.2.1.55) gene encoding alpha-L-arabinofuranosidase, which are related to odorless non-volatile glycosides and glycosidase activities that contribute to the wine aroma, were detected in the genome of *E. faecium* 1702. Finally, the *estA* gene encoding a putative tributyrin esterase (ko:K03930) linked to increased esters that contributed to the wine’s fruity aromas was also detected in the genome. It is interesting to note that several metabolic pathways and gene clusters detected in this genome are under investigation and will be reported in separate paper (s).

#### 3.11.3. Bioremediation of Pollutant Compounds

The strain harbored the *azoA* gene encoding the AzoR azoreductase (AzoR) (K01118). This enzyme is responsible for sulfonated mono-azo dye (Methyl orange) and di-azo dye (Congo red) degradation. Azo compounds date back to 1859, and the high reactivity of -N=N- double bonds endows azo compounds with wide applications in many fields such as organic dyes and radical reaction initiators.

## 4. Discussion

### 4.1. Genomic Traits of E. faecium 1702

The size of the chromosome of *E. faecium* 1702 is 2,621,416 bp, featuring a G + C content of 38.03%. Several studies have reported similar chromosome sizes in *E. faecium* isolates, such as 2,689,107 bp (GC 38.1%) [74], 2,693,877 bp (GC 38.4%) [75], 2,935,283 bp (GC 38.0%) [76], and 2,971,195 bp (GC 37.97%) [77]—in general, ranging from 2.6 Mbp to 2.9 Mbp [78]—however, bigger chromosomes have also been reported, such as 3,597,440 bp (GC 37.94%) [79]. The GC% content is also similar to that reported in *E. faecium* (37.7 to 38.46%) [80].

In the strain, there are 2413 CDSs out of 2521 predicted chromosomal genes, 24 RNAs (16S rRNA (*n* = 8), 5S rRNA (*n* = 4), 23S rRNA (*n* = 4)), 60 tRNA, and 19 sRNA (*n* = 19). This finding is similar to the genetic traits of the commercially available probiotic strain *E. faecium* T-110 (NZ_CP006030.1) containing 2639 genes with 2502 CDCs, 54 pseudogenes, 18 rRNAs [5S (*n* = 6), 16S (*n* = 6), 23S (*n* = 6)], and 65 tRNAs [75]. Similarly, the *E. faecium* AB157 strain, an effective GABA-producing strain from Sichuan Paocai, harbors 2446 chromosomal genes, including 2271 CDSs, 60 tRNAs, 10 rRNAs, and 4 non-coding RNAs (ncRNAs) [80]. Despite the few differences exhibited by the aforementioned strains, Ghattargi et al. [81] showed no significant differences (*p*-value ≤ 0.05, Kruskal–Wallis test) between groups of probiotic, non-pathogenic non-probiotic (NPNP), and pathogenic *E. faecium* strains with respect to their genome size, G + C content, average number of genes, and coding DNA sequences (CDSs)—however, they showed that the average numbers of annotated protein-encoding genes were 2570, 2639 and 3093 for the probiotic, NPNP, and pathogenic groups, respectively.

The strain belongs to the clonal lineage ST722, which is not known as a high-risk clonal lineage (HRCL). This ST was previously reported in two bacteriocinogenic *E. faecium* strains isolated from poultry meat samples collected from local markets in La Rioja, Spain [82].

### 4.2. Soft Features

*E. faecium* strain 1702 harbored several genes encoding putative virulence factors. Those genes encode (i) biofilm formation (*sag-Entfm-hosp*), (ii) proteins involved in surface adhesion to the extracellular matrix (*acm-Entls*, *scm-Entfm-hosp*, *fnm-Entfm-com*, *sgrA-Entfm-hosp*, *fms11Entfm-hosp*, *fms13-Entfm-hosp*, *fms14-Entfm-hosp*, *fms15-Entfm-hosp*, *fms16-Entfm-hosp*, *fms17-Entfm-hosp*, *fms19-Entfm-hosp*, *empA-Entfm-hosp*, *empB-Entfm-hosp*, *empC*-*Entfm-hosp*), (iii) carbohydrate metabolism and cell growth (*ccpA-Entfm-com*), (iv) general stress proteins important in bile salt tolerance and general intestinal adaptation (*gls20-Entfm*, *gls33-Entfm, glsB-Entfm*, *glsB1-Entfm*), and (v) phosphotransferase systems important in carbohydrate transport (*bepA-Entfm-com*) [83,84]. However, the presence of those specific virulence factors does not mean that the harboring organism may be harmful. Indeed, it is well known that adhesion and capsule formation are essential even for commensal and probiotic enterococci to persist, promote colonization, and avoid elimination from the host. Moreover, genes involved in adhesion and capsule formation have been identified among enterococci designated as starter/probiotic strains [85]. Taken together. the aforementioned genes, especially those related to cell surface adhesin and collagen-binding proteins, promote cell adhesion and colonization of the gut [80,86]. According to the European Food Safety Authority [87], the presence of the *IS16*, *esp* (enterococcal surface protein), *gelE* (gelatinase), *asa1* (Aggregation substance), *cyl* (Cytolysin), *hyl* (hyaluronidase-like protein), and hemolysin (*hla*, *hlb*, *hld*, *hlg*) genes in the enterococi genome is considered unacceptable, and any strain containing such genes cannot be regarded as safe. Notably, *E. faecium* 1702 lacks these virulence-associated genes that are commonly found in pathogenic *E. faecium* strains, thereby supporting its safety profile [80,88].

Antimicrobial-resistant *E. faecium* strains have been increasingly reported worldwide. From a clinical point of view, in *E. faecium*, alarming phenotypes of resistance concern beta-lactams, aminoglycosides (high-level resistance), glycopeptides (vancomycin and teicoplanin), and oxazolidinones (linezolid and tedizolid) [89]. *E. faecium* 1702 was not resistant to the aforementioned antibiotics, suggesting the possibility of synergistic bactericidal effects of these drugs. The strain was phenotypically resistant to rifampicin, ciprofloxacin, trimethoprim, and trimethoprim-sulfamethoxazole, which are clinically insignificant, because it is not the preferred treatment for enterococcal infections.

The genomic analysis showed the presence of *aac(6′)-Ii* and *msrC* genes encoding the aminoglycoside N-acetyltransferase AAC(6′)-Ii (mediating resistance to tobramycin, kanamycin, and amikacin) and the ABC-F-type ribosomal protection protein Msr(C) related to macrolide, lincosamide, and streptogramin resistance (low-level resistance to erythromycin and quinupristin), respectively [86]. Genes encoding resistance to ciprofloxacin and erythromycin are clinically insignificant since both antibiotics are not among the preferred treatments for enterococcal infections. Therefore, both resistance determinants (*aac(6′)-Ii* and *msrC*) are of no clinical significance and they are also considered intrinsic for the *E. faecium* species. Moreover, they both have known chromosomal locations that significantly decrease the risk of horizontal gene transfer to other bacteria via conjugation [90]. Interestingly, the strain harbored two multidrug efflux ABC transporters encoded by the *efmA* gene responsible for macrolides and fluoroquinolone resistance and the *efrA* gene leading to resistance toward macrolide antibiotics, fluoroquinolones, and rifamycin [86,91]. Furthermore, it harbored the *fosI* gene encoding fosfomycin resistance as well as the intrinsec ABC-F-type ribosomal protection-like protein Eat(A) responsible for the lincosamides, streptogramins A, and pleuromutilins resistance. Globally, at least ten *fos* genes with various variants (*fosA*, *fosB*, *fosC*, *fosD*, *fosE*, *fosG*, *fosH*, *fosK*, *fosI*, *fosX*) encoding enzymatic deactivation by fosfomycin glutathione *S*-transferase enzymes have been reported [92]. Those genes are mainly plasmid-borne and frequently found as gene cassettes in integrons, which enhance their spread. The *fosI* gene, carried by a class 1 integron on the pBRA100 (56,265 bp) plasmid, was firstly identified in *Mycobacterium abscessus* strain F1725 [92] and then after was also reported in other species such as *Salmonella* spp. and *Escherichia coli* strains [93].

Pleuromutilins, inhibitors of protein synthesis by interacting with the 30S subunit of ribosomes, were discovered as natural agents in 1951. The C-14 derivatives tiamulin (TIA) and valnemulin are widely used in veterinary medicine, and retapamulin (RET) and lefamulin (LEF) are approved for human use against methicillin-resistant *S. aureus* or beta-lactamase-producing streptococci [94]. There is growing evidence that Gram-positive infections are becoming more resistant to pleuromutilins; in particular, most isolates demonstrate the PLSA phenotype by being cross-resistant to pleuromutilins, lincosamides, and streptogramins A. In *E. faecium*, the *eatA* gene encoding Eat(A) is an intrinsic gene commonly reported by WGS as well as the intrinsic genes *efmA* and *efrA* [86,91,95]. Other intrinsic genes, in part mentioned above, linked to antimicrobial resistance have been reported in *E. faecium*, such as *efrB* (resistance to macrolide antibiotic, rifamycin antibiotic, fluoroquinolones) and *efmM* (aminoglycoside) [91]. Taken together, the strain harbored intrinsic resistance genes that are not considered ‘harmful’ genes. Indeed, fortunately, the strain was free of acquired antimicrobial resistance genes, including those conferring resistance to clinically critical antibiotics such as glycopeptides (vancomycin and teicoplanin; *vanA*, *vanB*), oxazolidinones (linezolid and tedizolid; *optrA*, *poxtA*, *cfr*), and high-level aminoglycosides (*aac(6′)-aph(2″)*). [96]. Even though the strain was phenotypically susceptible to vancomycin, the genome contained two copies of the *vanY* gene in the *vanB* cluster, which normally confer resistance to vancomycin. Indeed, the *vanB* cluster mainly contains *vanY* in association with other genes (*vanR_B_-vanS_B_-vanY_B_-vanW-vanH_B_-vanB-vanX_B_*). However, in the absence of the remaining components of the *vanB* cluster, *vanY* is unable to confer vancomycin resistance since it is not required for, but contributes to, high-level resistance to vancomycin. Similar findings have previously been reported in enterococci and staphylococci as well [97]. VanY is a membrane-associated D,D-carboxypeptidase that hydrolyzes the C-terminal D-Ala or D-Lac residue of peptidoglycan precursors but lacks transpeptidase activity [98];d therefore, its occurrence in the absence of the entire *vanB* cluster indicates simply its role in peptidoglycan synthesis and is not linked to vancomycin resistance.

Mobile genetic elements such as plasmids, transposons, and phages are the main genetic elements driving antibiotic resistance spread within bacteria. In addition, insertion sequences (ISs) have proven to play a crucial role in the transposition phenomenon of resistance and virulence genes as well. In addition, by changing their position in the genome, they play critical roles in genome function and evolution. The analysis revealed the presence of the IS*Efa10* gene coding for an IS3 family transposase as well as a *TnpB*-related gene coding for an RNA-guided endonuclease TnpB family protein. IS*Efa10* is similar to other transposases and integrases of *Enterococcus* (GenBank accession numbers ZP05663216 and ZP05674394). In enterococci, various ISs have been reported, such as IS*Ef1*, IS*256*, IS*Efa7*, IS*Efa8*, IS*1216*, IS*1297*, IS*1476*, IS*1252*, IS*1062*, IS*Aac3*, and IS*Lmo3*, either in association with antibiotic resistance genes or not [99,100].

It is worthy to note that the strain was free of any plasmid; this might explain in part the absence of acquired antibiotic resistance genes. In bacteria, several mechanisms control the acquisition and spread of plasmids. The CRISPR and CRISPR-Cas systems provide adaptive immunity against invading mobile genetic elements (MGEs), such as plasmids and phages. The *E. faecium* 1702 genome did not contain a CRISPR and CRISPR-Cas system (CRISPR)-consensus sequence.

Biogenic amines (BAs) such as putrescine, cadaverine, spermidine, tryptophan, 2-phenylethylamine, spermine, histamine, serotonin, tyramine, trimethylamine, dopamine, and agmatine, are nitrogenous compounds with a low molecular weight that are created in foods by microbial decarboxylation of the source amino acids [101]. The accumulation of BAs in foods represents a critical issue in food safety and spoilage assessment. Ingestion of foods containing elevated concentrations of BAs has been associated with adverse toxicological responses, leading to various forms of foodborne intoxication manifested by symptoms such as cephalalgia, hypotension, cardiac palpitations, peripheral edema, emesis, and diarrhea [101]. Tyramine is the most prevalent biogenic amine, detected in cheese and fermented meat products, predominantly produced by LABs belonging to the genera *Enterococcus* and *Lactobacillus*. Certain strains of *E. faecalis*, *E. faecium*, and *E. durans* are thought to be extremely potent tyramine producers. Putrescine production has been reported in some LAB strains, including *Lactobacillus brevis* and *Lactobacillus hilgardii* recovered from wine and *Lactobacillus curvatus*, *E. faecalis*, *Lactobacillus fermentum*, and *Lactobacillus paracasei* isolated from cheese, beef, and sausage [102,103]. A search of the *E. faecium* 1702 genome for genes associated with the production of BAs, including genes coding for the enzymes histidine decarboxylase, tyrosine decarboxylase, ornithine decarboxylase, lysine decarboxylase, and agmatine deiminase, which are responsible for the synthesis of BAs in lactic acid bacteria [104], showed an absence of all of them. This is an important trait of this strain if used as a food conserver or as a starter in different foodstuffs (meat, dairy products, vegetables, and wine).

### 4.3. Probiotic Properties

The genus *Enterococcus* produces extracellular antimicrobial proteins known as enterocins, which are ribosomally synthesized. These compounds serve as a defense mechanism, inhibiting the growth of competing bacteria and thereby enhancing the competitiveness of bacteriocin-producing strains. Numerous enterocins have been identified in enterococci from diverse sources and hosts, including *entA*, *entB*, *entQ*, *entX*, *entAS-48*, *entF4–9*, *entL50*, *entEJ97*, *entRJ-11*, *entE-760*, *ent416K1*, and *entKT2W2G*, among others [105]. The *E. faecium* 1702 strain demonstrated strong inhibitory activity against *Listeria monocytogenes* as well as other *E. faecium* strains, which was attributed to the production of proteinaceous substances. Genome analysis revealed the presence of *entA*, *entB*, and *entX*, encoding enterocin A, enterocin B, and enterocin X, respectively. As enterocins are recognized as promising natural biopreservatives, their production by *E. faecium* 1702 may confer beneficial properties by inhibiting undesirable bacteria, including food spoilage organisms and pathogens [106]. The presence of *entA*, *entB*, and *entX*, either individually or in combination with other enterocins, has been reported in enterococcal strains from human, animal, clinical, food, agricultural, and environmental sources [107,108]. For instance, the multi-enterocin-producing *E. faecium* EFD strain, isolated from fresh pollen granules, harbors five bacteriocins: bacteriocin 32, enterocin A, enterocin P, enterocin SE-K4, and enterolysin A [109]. Several studies have suggested a correlation between the presence of known enterocin genes and the antagonistic spectrum of enterococcal isolates, although such a relationship has not been consistently observed in all strains [107,110]. While the presence of multiple bacteriocin genes may indicate the potential for a potent antimicrobial mixture, it does not necessarily guarantee that all encoded bacteriocins are actively produced [111]. Enterocin B belongs to class IId, comprising non-pediocin-like, single-peptide bacteriocins, and shows strong homology to carnobacteriocin A. It exhibits activity against a broad range of pathogens, including *Listeria monocytogenes*, *Staphylococcus aureus*, and *Acinetobacter baumannii*, and has been reported to inhibit biofilm formation as well as demonstrate activity against human cancer cell lines [105]. Enterocin X, initially identified in *E. faecium* and later found in *Leuconostoc* species isolated from the traditional Korean fermented vegetable kimchi [112], is a class IIb bacteriocin composed of two complementary peptides, X-alpha and X-beta (Xα and Xβ), each with distinct antimicrobial properties [113]. Enterocin X mainly exhibits a narrow spectrum of weak to moderate antibacterial activity. The structural genes for enterocin B and enterocin X are located in close proximity within the genome. The enterocin B gene cluster includes a Lactococcin-G-processing and transport ATP-binding protein (*lagD*) and a putative UV-damage repair protein (*UvrX*). A similar gene organization has been reported in *E. faecium* R9, which carries enterocin B, enterocin X, and enterocin P [79], as well as in *E. faecium* NT04 [114]. Enterocin A shares sequence homology with class IIa, pediocin-like bacteriocins, which are characterized by strong antilisterial activity [105,115]. The *entA* cluster in *E. faecium* 1702 exhibits a genomic structure highly similar to that reported in other strains, including *E. faecium* NT04, isolated from the oral microbiota of healthy Egyptian adults [114]; *E. faecium* CTC492, from fermented Spanish sausage [115]; and the dairy-associated strain *E. faecium* DPC1146 [116].

The *E. faecium* 1702 genome encodes 87 CAZymes distributed across four major classes (GH, GT, CE, and PL), all of which play key roles in carbohydrate processing. The diversity of these CAZyme families indicates a robust capacity for carbohydrate metabolism, supporting efficient energy production and bacterial growth. Such metabolic versatility may provide advantages for bioprocessing, fermentation, and the development of functional probiotic formulations. GTs, in particular, participate in the biosynthesis of disaccharides, oligosaccharides, and polysaccharides by catalyzing the formation of glycosidic bonds. The strain’s broad carbohydrate utilization profile is therefore an important indicator of its functional potential and provides a foundation for informed strain selection and cultivation strategies. Notably, carbohydrate-binding modules (CBMs) and auxiliary activity (AA) families were not detected, consistent with previous observations that GH, GT, and CE families predominate over other CAZyme categories, such as CBMs, AAs, LPs, Dockerin domains, S-layer-binding modules (LSH), and cohesin domains, in various LAB strains [117,118]. Taken together, the analysis identified 326 genes related to carbohydrate metabolism and transport, as annotated in the COG and KEGG pathways. Of these, 26.68% were associated with carbohydrate-active enzymes (CAZymes). In LABs, well-known carbohydrates are metabolized into short-chain fatty acids (SCFAs), which serve as an additional energy source for the host. SCFAs play a vital role in gut health [119] and contribute to the microbiota–gut–brain axis [120]. GHs are widely distributed across prokaryotic, eukaryotic, and archaeal species, reflecting their fundamental importance in carbohydrate degradation. They constitute a broad repertoire of cell wall-degrading enzymes that hydrolyze glycosidic bonds either between two or more carbohydrate units or between a sugar and a non-sugar moiety within carbohydrates or oligosaccharides [121,122,123]. To date, 189 glycoside hydrolase (GH) families have been identified (http://www.cazy.org/Glycoside-Hydrolases.html; accessed on 10 January 2025). While starch and low-molecular-weight sugars can be fully digested and absorbed, oligosaccharides and polysaccharides remain indigestible in the small intestine because monogastric animals lack the necessary enzymatic machinery. Consequently, a complex mutualistic relationship has evolved between mammalian hosts and their gut microbiota, which supply the enzymes required to access the abundant energy stored in these otherwise indigestible carbohydrates. Interestingly, the CAZyme analysis revealed GHs as the dominant class of enzymes involved in carbohydrate metabolism in *E. faecium* 1702, underscoring their importance in supporting its potential as a probiotic candidate. Among the 58 GHs identified, distributed across 22 families, the most abundant were GH1 (β-glucosidases) (*n* = 18), GH123 (β-N-acetylgalactosaminidases) (*n* = 8), GH43 (α-arabinofuranosidases) (*n* = 5), GH73 (peptidoglycan endo-β-1,4-N-acetylglucosaminidases) (*n* = 5), and GH13 (α-glucosidases) (*n* = 5). Enzymes encoded by the GH1, GH13, and GH43 families, such as α-amylase, maltodextrinase, cellulase, and hemicellulase, expand the strain’s capacity to degrade diverse carbon sources, thereby enhancing its metabolic adaptability and environmental fitness. For example, enzymes involved in arabinan degradation, responsible for releasing arabinosyl oligomers and L-arabinose, were identified in *E. faecium* 1702, including genes encoding α-L-arabinofuranosidases (EC 3.2.1.55) and endo-α-1,5-L-arabinanases (EC 3.2.1.99), which are typically distributed across GH families 3, 43, 51, and 62. Notably, GH73 family enzymes cleave the β-1,4-glycosidic linkage between N-acetylglucosamine (NAG) and N-acetylmuramic acid (NAM) in bacterial peptidoglycan; due to this specificity, they are often classified as β-N-acetylglucosaminidases. Some GH73 enzymes, such as lysozyme, may also exhibit antimicrobial activity [123]. Several minor GHs were also detected, including β-galactosidases from GH2 (*n* = 2) and 6-phospho-N-acetylmuramidases from GH170 (*n* = 3). Enzymes such as β-glucosidase (GH1), α-glucosidase (GH13_23), and β-galactosidase (GH2) contribute to the breakdown of carbohydrates like sucrose, lactose, and various oligosaccharides. These metabolic capabilities are essential for the growth and functional performance of the strain in diverse environments, including dairy products [124]. Moreover, β-galactosidases and the products of their activity (lactose hydrolyzed into glucose and galactose) are widely applied in the dairy industry for the breakdown of lactose in milk and whey [123]. This process facilitates digestion, particularly for lactose-intolerant individuals, meaning that β-galactosidase-producing strains can help alleviate symptoms of lactose intolerance.

In addition, CAZyme analysis further identified 18 enzymes belonging to five glycosyltransferase (GT) families (GT2, GT4, GT8, GT28, GT51) among the 138 known GTs (https://www.cazy.org/GlycosylTransferase-family; accessed on 10 January 2025). Of these, eleven enzymes were classified as GT2, representing 61.1% of the GTs. GTs are responsible for the synthesis of sucrose, cellulose, chitin synthase, as well as glucosyl- and galactosyltransferase activities. They also play a key role in the biosynthesis of exopolysaccharides, which influence the texture of fermented products and confer prebiotic, immunomodulatory, antioxidant, and other beneficial effects in dairy foods [125]. Furthermore, members of the GT2, GT4, and GT8 families are primarily involved in lipopolysaccharide (LPS) biosynthesis, biofilm formation, and the production of extracellular and capsular polysaccharides [126]. In contrast, members of the GT28 and GT51 families are mainly associated with peptidoglycan synthesis, where they contribute to the maintenance of cell wall integrity [126].

Another class of enzymes identified in the CAZyme analysis was the CEs. CEs facilitate the hydrolysis of complex polysaccharides by glycoside hydrolases, catalyzing the de-O- or de-N-acetylation of glycans and substituted saccharides. To date, 20 CE families have been characterized (http://www.cazy.org/Carbohydrate-Esterases.html; accessed on 16 January 2025). In the *E. faecium* 1702 strain, ten predicted CE genes were assigned to six families: CE1, CE4, CE8, CE9, CE12, and CE19. The CE4 family was represented by three enzymes encoding peptidoglycan N-acetylglucosamine deacetylases (EC 3.5.1.104), while CE1 and CE12 encode acetyl xylan esterases (AcXEs; EC 3.1.1.41). Both CE8 and CE19 enzymes encode pectin methylesterases (or pectinesterase) (EC 3.1.1.11), whereas CE9 encodes N-acetylglucosamine 6-phosphate deacetylase (EC 3.5.1.25). Most of these CEs play critical roles in bacterial peptidoglycan recycling, amino sugar metabolism, and the degradation of plant polysaccharides. CE1 constitutes the largest esterase family, with 5062 entries listed in the CAZy database, and its members primarily target xylan. In contrast, CE19 family members are mainly involved in pectin degradation. Carbohydrate esterases have shown considerable potential for industrial applications, including in the food, pulp and paper, biofuel, animal feed, pharmaceutical, and medical sectors [127].

The cholesterol-lowering ability of probiotic bacteria, reflected by reductions in total cholesterol and LDL levels, is an important trait that supports their survival and colonization in the lower intestine. This property is mediated by bile salt hydrolase (BSH) activity, which modulates bile acid metabolism and the enterohepatic cycle. According to the FAO/WHO Guidelines for the Evaluation of Probiotics in Food, BSH activity, encoded by choloylglycine hydrolase (EC 3.5.1.24), is recognized as an important functional criterion for selecting probiotic strains [8]. In *E. faecium* 1702, the gene encoding choloylglycine hydrolase was identified, suggesting its potential contribution to cholesterol metabolism. Similar cholesterol-lowering traits have been reported in other *E. faecium* strains. For example, *E. faecium* GEFA01 reduced total cholesterol and LDL in mice fed a high-fat diet, likely via bile salt deconjugation and modulation of the gut microbiota [128]. Strain *E. faecium* YY01 displayed in vitro cholesterol degradation and harbors a BSH-encoding gene confirmed by genome sequencing [129]. Additionally, *E. faecium* LR13 and related isolates demonstrated strong in vitro cholesterol-lowering activity coupled with the presence of the *bsh* gene [130]. The malolactic enzyme gene (*mleS*) can indirectly support bile tolerance in *E. faecium* by converting L-malate to L-lactate while consuming intracellular protons, thereby helping to maintain the cytoplasmic pH during bile-induced acid stress. Similar roles of the malolactic pathway in acid and bile adaptation have been reported in LABs, where malolactic enzyme activity enhances intracellular pH homeostasis and overall gastrointestinal survival [131].

A critical trait for probiotic development is the ability to withstand and adapt to the harsh conditions of the gastrointestinal tract of humans and animals, including temperature fluctuations, pH stress, and oxidative or osmotic challenges. In the genome of *E. faecium* 1702, we identified a general stress response protein encoded by the *usp* gene, along with a set of proteases and chaperones that likely contribute to heat-shock tolerance. These include ATP-dependent proteases such as GrpE, GroES, GroEL, CtsR, HslV, HslU, ClpB, ClpC, ClpE, ClpP, and ClpX, as well as the molecular chaperones DnaK and DnaJ [132]. Notably, ClpB, ClpC, and ClpE have been implicated in thermotolerance in other Gram-positive bacteria, supporting the role of these genes in the heat stress response. The presence of the groES-groEL, dnaJ-dnaK, and GrpE–HrcA chaperone complexes, as well as the HslO/HslU/HslV protease system, further underlines the capacity of *E. faecium* 1702 to mount a robust heat-shock response [132,133]. Several studies have highlighted the advantages of heat-resistant strains, including faster growth at elevated temperatures, lower susceptibility to phage infection, accelerated lactate production, and reduced formation of unwanted by-products. Consequently, strains with enhanced thermal tolerance are of considerable interest to various industrial applications [134,135]. In *Enterococcus* species, heat resilience is a well-documented phenomenon. For example, previous research demonstrated that *E. faecium* isolates exhibit significant tolerance to heat exposure, likely aided by stress response systems [136]. Moreover, proteomic analyses of *E. faecium* persister cells under stress conditions revealed the upregulation of stress-associated proteins including ClpX, further indicating the importance of proteases and chaperones in stress survival [137]. Genetic studies have also confirmed that the *usp* gene contributes to long-term survival under starvation in *E. faecium* (strain E745), highlighting its role in stress resilience [138]. Finally, adaptive heat tolerance has been observed in the *E. faecium* RS047-wl strain exposed to sublethal heat, where enhanced expression of membrane- and cell-wall-stabilizing systems (e.g., fatty acid metabolism, peptidoglycan restructuring) was correlated with better survival [139].

Resistance to gastrointestinal pH stress is critical for the probiotic potential of any strain. In *E. faecium* 1702, we identified eight genes encoding ATP synthase subunits, in addition to the *nhaA*, *nhaC*, and *nhaP1* genes, which encode Na (+)/H (+) antiporters NhaA, NhaC, and NhaP1, respectively. These ion transport systems are known to contribute to acid tolerance in enterococci and other bacteria [140]. Furthermore, the genome harbors genes for alkaline-shock proteins (*asp1*, *aspS*, WQ51_04310, *yloU*), as well as ATP-binding cassette (ABC) transporters involved in osmotic stress adaptation (such as *opuAA*, *opuCA*, *opuCB*), which facilitate the uptake and accumulation of osmoprotectants. In addition, we detected genes implicated in the oxidative stress defense (including *npr*, *nox*, *trxB*, *mntA*, *mntB*, *mntC*). Finally, several genes related to bile tolerance were present, such as *ppaC*, *cfa*, *glnA*, and *arsB*, among others. These genetic determinants reflect a multifaceted stress resistance profile, likely enhancing the survival and persistence of *E. faecium* 1702 in the gastrointestinal tract. Similar adaptations have been documented in other *E. faecium* strains. For instance, a functional genomic study of bile salt resistance in *E. faecium* E1162 identified a variety of genes, including ion transporters, general stress proteins, and two-component systems contributing to survival under bile stress [141]. *MurE* and *MurF* encode cytoplasmic ligases that catalyze key steps in peptidoglycan (PG) precursor synthesis (MurE adds a peptide moiety; MurF adds the terminal D-Ala-D-Ala dipeptide). However, in *E. faecium*, high-throughput genetic screens for bile salt-sensitive mutants (M-TraM) did not recover transposon insertions in *murE* or *murF* as significantly contributing to bile resistance, suggesting they may not play a specialized adaptive role under bile stress. Thus, while PG synthesis is essential for cell integrity (and indirectly may influence stress survival), *murE* and *murF* do not appear to function as specific bile resistance determinants in *E. faecium* [141].

In addition to the aforementioned traits, the genome of *E. faecium* 1702 contains several genes that likely enhance intestinal adhesion, including fibronectin-binding protein A, enolase, lipoprotein signal peptidase II (*lspA*), elongation factor Tu (*tuf*), and LPXTG-motif cell wall-anchored proteins (sortases), supporting its high adhesive potential. Sortase enzymes, in particular, mediate the covalent attachment of sortase-dependent proteins to the cell wall of Gram-positive bacteria and have been associated with key probiotic attributes, including adhesion to host tissues, maintenance of the mucus barrier, and modulation of immune signaling [142]. Several commercially available strains also produce sortase. For instance, *E. faecium* T-110, a component of the probiotic consortium BIO-THREE widely used in humans, animals, and aquaculture, expresses sortase [75]. Similarly, the urolithin A-producing *E. faecium* FUA027, isolated from human fecal samples, harbors multiple genes involved in adhesion and colonization, including adhesion lipoprotein, S-ribosylhomocysteine lyase (luxS), and segregation and condensation protein B (*scpB*) [143]. Elongation factor Tu (EF-Tu) is a canonical cytosolic translation factor, but in several bacteria it “moonlights” on the cell surface, mediating adhesion to host molecules such as mucins and fibronectin [144]. In *Lactobacillus* species and some pathogens, surface-associated EF-Tu has been experimentally shown to bind these host factors, contributing to colonization [145]. However, in *E. faecium*, there is currently no direct evidence that EF-Tu is surface-exposed or functions as an adhesin. Therefore, the presence of EF-Tu in the genome only indicates a potential role in adhesion, and functional validation, such as surface proteomics, immunolocalization, or adhesion assays, is required to confirm its involvement. This cautious interpretation ensures that claims about EF-Tu’s role in *E. faecium* adhesion remain scientifically sound.

Phytase is another beneficial enzyme predicted in *E. faecium* 1702. Phytate (myo-inositol hexakisphosphate, InsP_6_) is the primary storage form of phosphorus in plant-based foods (such as cereals and legumes). However, it is also a well-known anti-nutrient because it chelates dietary minerals (e.g., Fe^2+^/Fe^3+^, Ca^2+^, Mg^2+^, Zn^2+^, Cu^2+^) and proteins, thereby reducing their bioavailability in the human and animal gut [146]. Since humans and monogastric animals lack endogenous phytase, the ability of a probiotic strain to produce phytase significantly increases its value; it can hydrolyze phytate, releasing bound phosphates and improving mineral absorption. However, we did not find clear genomic evidence in the literature for the phytase gene in *E. faecium*. While some probiotic LABs are known to produce phytase, reports of *E. faecium* phytase production are limited. For example, a study of *E. faecium* A86 showed phytate-degrading activity in vitro [147]. Additionally, in a poultry study, *E. faecium* supplementation was associated with improved phosphorus absorption, which the authors hypothesized could stem from either direct phytase production by *Enterococcus* or enhanced activity of other microbial phytases in the gut [148]. Given the limited and indirect evidence, phytase production by *E. faecium* 1702 is a predicted trait, and future work (e.g., biochemical assays or transcriptome analysis) is needed to validate true phytase activity and confirm the presence of a functional phytase gene.

The strain possessed 55 metabolic genes associated with the biosynthesis of a wide range of cofactors and vitamins. Notably, it encoded complete or near-complete pathways for several B-group vitamins, including biotin, folate, pyridoxine, riboflavin, and thiamine, as well as enzymes involved in the synthesis of menaquinone (vitamin K_2_) and phylloquinone (vitamin K_1_). These vitamin-producing capacities are highly desirable traits for a probiotic strain. B-vitamins and vitamin K play essential roles in fundamental cellular functions such as DNA replication, repair, and methylation, as well as amino acid and nucleotide biosynthesis, and similar biosynthetic capabilities have been documented in many lactic acid bacteria [149]. Comparable traits have also been reported in other *E. faecium* genomes: for example, *E. faecium* NT04 has been shown by WGS to carry folate biosynthesis genes [114]. Additionally, comparative genomics of *E. faecium* 17OM39 and the marketed probiotic strain E. faecium T110 revealed complete pathways for folate and thiamine biosynthesis [81]. Moreover, E. *faecium* LZ86 was reported to produce biologically active adenosyl-cobalamin (vitamin B_12_), which supports its probiotic potential [150]. Since humans cannot synthesize 13 essential vitamins, microbial production of these micronutrients represents an important exogenous source. Therefore, the metabolic profile of *E. faecium* 1702 underscores its potential for biotechnological applications in vitamin production and as a probiotic strategy to mitigate micronutrient deficiencies.

In our strain, we detected several genes involved in heavy-metal uptake and tolerance/resistance, including determinants mediating resistance to zinc, cobalt, cadmium, and arsenate. This suggests that the strain could survive in heavy-metal-rich environments via mechanisms such as efflux pumps (e.g., for zinc, cobalt, cadmium) and sequestration (e.g., arsenate), and it may also mitigate the toxic impact of these compounds within the host gut (particularly through arsenate sequestration). Heavy metals enter animal diets not only as contaminants but also, paradoxically, as essential nutrients [151,152]. Indeed, essential trace elements like zinc and cobalt are routinely added as feed supplements to improve productivity and animal health, but at elevated concentrations, these same metals can become toxic. Over the past decade, various LABs, including *Lactobacillus acidophilus*, *L. plantarum*, *L. reuteri*, *L. rhamnosus*, *L. casei*, and *L. brevis*, have been reported to chelate and detoxify heavy-metal ions such as Hg^2+^, Pb^2+^, Cd^2+^, and Al^3+^ [153]. Kinoshita et al. [154] demonstrated that LABs can bind heavy metals and suggested that oral administration of these bacteria may significantly reduce intestinal absorption of heavy metals. Similarly, Topcu and Bulat [155] investigated *E. faecium* EF031 and *E. faecium* M74, observing that EF031 and a probiotic culture of M74 effectively removed cadmium and lead from the liquid medium. Importantly, *E. faecium* strains with comparable heavy-metal resistance genomic traits have been described. For instance, *E. faecium* Am5, isolated from the gut of *Apis mellifera*, harbors the *czcD* gene (conferring cobalt/zinc/cadmium resistance), the *cadA* ATPase (cadmium exporter), and a *zur* regulator for zinc uptake; its genome also carries *copB*, a copper-translocating ATPase [156]. Another strain, *E. faecium* CX 2–6, isolated from a heavy-metal-contaminated farmland, has been fully sequenced; under cadmium stress, about 47% of its genes, including many core genes, show differential expression, and it appears to reorganize its transcriptional and metabolic architecture to cope with cadmium [157]. In broader surveys, comparative genomics across hundreds of *Enterococcus* isolates also revealed that metal tolerance operons (e.g., *arsA* for arsenate, *merA* for mercury, *tcrB* for copper) have been widely distributed in *E. faecium* populations over the last century, often co-localized with antibiotic resistance genes [158]. Thus, the presence of heavy-metal tolerance genes in our strain not only supports its ecological robustness but also aligns with a growing body of evidence from other *E. faecium* isolates. This makes our strain a promising candidate both for survival in challenging environments and for reducing host exposure to toxic metals.

### 4.4. Pharmaceutical Properties

Cancer cells often lack certain amino acid biosynthetic enzymes, rendering them auxotrophic for these amino acids, whereas normal cells retain these pathways. This metabolic difference provides a therapeutic window exploited in cancer treatment. Several amino acid-degrading enzymes, including arginine deiminase, L-methionase, L-arginase, lysine oxidase, L-glutaminase, and L-phenylalanine, are currently under clinical evaluation, while L-asparaginase has been approved as an effective therapy against acute lymphoblastic leukemia (ALL) and other auxotrophic malignancies [159]. In our study, *E. faecium* 1702 harbored the ansB, *glsA*, and *arcA* genes, encoding L-asparaginase, L-glutaminase, and arginine deiminase, respectively. L-asparaginase, the first bacterial enzyme approved for cancer treatment, catalyzes the hydrolysis of L-asparagine to L-aspartate and L-glutamine. Tumors auxotrophic for asparagine, due to low or absent asparaginase expression, are particularly sensitive to this treatment. By depleting plasma asparagine levels, L-asparaginase effectively starves tumor cells, inducing apoptosis [159], and is widely used in pediatric ALL therapy [159]. L-glutaminase (also known as L-glutamine amidohydrolase) has attracted interest for diverse biological activities. For example, L-glutaminase from *Bacillus cereus* MTCC 1305 exhibits anti-tumor activity against hepatocellular carcinoma (Hep-G2), while the enzyme from *Alcaligenes faecalis* is active against HeLa cells. L-glutaminase from *Pseudomonas 7A* has antiviral properties, whereas the enzyme from *Bacillus amyloliquefaciens* enhances food flavor, and *B. cereus* LC13’s enzyme shows antioxidant potential in combination with ascorbic acid [160]. Arginine deiminase, the third anticancer enzyme identified in our strain, targets arginine-auxotrophic tumors by hydrolyzing L-arginine to citrulline and ammonia. Arginine is a precursor for several key molecules, including nitric oxide, polyamines, nucleotides, proline, and glutamate, and it participates in biosynthetic pathways that influence carcinogenesis. Nitric oxide acts as a multifunctional messenger in tumor development, while proline can alter bioenergetics and tumor growth [161]. In adults, arginine is semi-essential, but it is essential for neonates. The urea cycle synthesizes arginine via argininosuccinate synthetase and lyase, converting L-citrulline and aspartic acid into L-arginine. Many tumors, including malignant melanoma and hepatocellular carcinoma, exhibit a deletion or downregulation of argininosuccinate synthetase, resulting in arginine auxotrophy. Arginine deiminase selectively starves these tumor cells, while normal cells remain unaffected due to endogenous arginine synthesis [159]. Arginine deiminase has been reported in multiple microbial sources, including *Bacillus pyocyaneus*, *Pseudomonas putida*, *Enterococcus* spp., *Halobacterium salinarium*, *Mycoplasma arginini*, *M. hominis*, *Pseudomonas aeruginosa*, *Lactococcus lactis* ssp. *lactis*, *P. furukawaii*, and *P. plecoglossicida* [162]. Some WGS and functional genomics studies have documented similar amino-acid-degrading genes in *E. faecium* strains. For example, *E. faecium* GR7 carries a fully characterized *arc* operon (including *arcA*, *arcB*, and *arcC*) encoding arginine deiminase and related enzymes [163]. Comparative genomics has also revealed a *glutaminase*-encoding gene (annotated as EFTG_00235) in *E. faecium*, which catalyzes the conversion of glutamine to glutamate and ammonia [164]. Moreover, the *arcA* gene from *E. faecium* SF68 was shown, via functional studies, to inhibit NF-κB signaling when expressed in a heterologous host, confirming the expression and activity of arginine deiminase in this probiotic strain [165].

The *E. faecium* strain 1702 was found to harbor genes encoding GABA production. Its genome contains the gad operon, comprising *gadB* (glutamate decarboxylase beta), *gadC* (glutamate/GABA antiporter), and *gadR*. In contrast, other studies have reported variations in the gad operon: in *Lactobacillus brevis* NCL912, it consists of *gadA* (glutamate decarboxylase alpha) and *gadC* [166], whereas *Levilactobacillus brevis* ILSH3 possesses two GAD-encoding genes, *gadA* and *gadB*, along with *gadC* and *gadR* [167]. Typically, bacteria that produce high concentrations of GABA carry either *gadA* or *gadB* in their genomes. Over the past several decades, GABA has been extensively studied in vertebrates due to its diverse physiological and pharmacological effects, including roles in brain development, analgesia, anxiolysis, hypotension, diuresis, and antidiabetic activity. Additionally, GABA functions as the principal inhibitory neurotransmitter in the mammalian central nervous system and can enhance plasma concentrations of growth hormones and protein synthesis in the brain [168,169,170,171]. Interestingly, the term “psychobiotics” has been proposed to describe a new class of probiotics capable of promoting mental wellness by influencing neurotransmitters and neuroactive compounds, including GABA, glutamate, serotonin, and brain-derived neurotrophic factor (BDNF) [170,171]. Given these properties, GABA is now widely used in pharmaceuticals and as a functional ingredient in foods, such as Gammalone, cheese, Gabaron tea, and Shochu. Consumption of GABA-enriched foods has been reported to inhibit cancer cell proliferation and improve memory and learning [166]. Moreover, its industrial applications have expanded recently, including as a precursor for the production of biodegradable Nylon 4 polymer [172]. Numerous LAB species and subspecies, such as *Streptococcus thermophilus*, *Lactobacillus paracasei*, *Levilactobacillus brevis*, and *Lactiplantibacillus plantarum*, are recognized as major GABA producers due to their high yields and established biosafety [168]. However, the potential of GABA-producing *Enterococcus* spp. remains significantly underexplored [173]. Detection of the gad operon in *E. faecium* 1702 further supports its probiotic potential. In the present study, GABA production was predicted solely by whole-genome sequencing; phenotypic and biochemical assays are needed to quantify actual GABA synthesis. Notably, *E. faecium* BS5, isolated from a dairy product, demonstrated high GABA production as measured by Thin-Layer Chromatography (TLC) and High-Performance Thin-Layer Chromatography (HPTLC) [174]. Similarly, the bacteriocinogenic *E. faecium* SH9 strain, isolated from marine shrimp, exhibited a strong GABA-producing ability and has been evaluated as a potential psychobiotic for the development of GABA-enriched functional products [173].

## 5. Conclusions

In conclusion, the multi-enterocin-producing *E. faecium* 1702 strain harbors a wide array of genes associated with probiotic traits, as well as metabolic capabilities relevant to biotechnological applications (e.g., cheese and wine production) and pharmaceutical use. The strain is considered safe, as it lacks virulence genes, acquired antimicrobial resistance determinants, and biogenic amine production. Taken together, these features suggest that *E. faecium* 1702 represents a promising probiotic candidate for both livestock and human applications. This study also highlights the value of probiogenomics as a powerful approach for the comprehensive characterization of probiotic strains and for uncovering metabolic pathways with potential biotechnological and pharmaceutical relevance. Several additional metabolic pathways were identified in *E. faecium* 1702, though they fall outside the scope of the present study and warrant future investigation.

## Figures and Tables

**Table 1:** General genome features of *E. faecium* strain 1702.

Feature of the Strain	Chromosome
Size (bp)	2,621,416
GC Content (%)	38.03
Gene Number	2521
Coding CDS Number	2413
transfer RNA Number	60
5S rRNA Number	4
16S rRNA Number	8
23S rRNA Number	4
sRNA Number	19
Plasmids	0
CRISPR	0
MLST Sequence Type	ST722

**Table 2:** Antibiotic resistance genes’ identification in the genome of *E. faecium* 1702.

Gene	Matching Region Identity (%)	Gene Product	Resistance Mechanism	Drug Class
*efmA* “AB1A69_00400”	99.77	Multidrug efflux MFS transporter EfmA	Antibiotic efflux	Macrolide antibiotic, fluoroquinolone antibiotic
*aac(6′)-Ii* “AB1A69_12035”	98.9	Aminoglycoside N-acetyltransferase AAC(6′)-Ii	Antibiotic inactivation	Aminoglycoside antibiotic
*msrC* “AB1A69_11335”	95.33	ABC-F-type ribosomal protection protein Msr(C)	Antibiotic target protection	Macrolide antibiotic, streptogramin antibiotic
*eatAv* “AB1A69_00840”	99.0	ABC-F-type ribosomal protection-like protein Eat(A)	Antibiotic target protection	Pleuromutilin antibiotic
*efrA* “AB1A69_08915”	82.81	Multidrug efflux ABC transporter subunit EfrA	Antibiotic efflux	Macrolide antibiotic, fluoroquinolone antibiotic, rifamycin antibiotic
*vanY* gene in *vanB* cluster	57.14		Antibiotic target alteration	Glycopeptide antibiotic
*fosI*	44.86		Antibiotic inactivation	Phosphonic acid antibiotic

**Table 3:** Virulence genes and their physiological functions in *E. faecium* 1702.

Virulence Gene	Alternative Names/VirulenceFinder Product	GGBK and NCBI Blast Search of the Same Sequences
*acm*-Entls	fms8 (MSCRAMM)/Adhesin of collagen from *E. faecium*	Collagen-binding MSCRAMM adhesin
*bepA*-Entfm-com	Biofilm and endocarditis-associated permease A/PTS transporter subunit EIIC	Fructose-specific PTS transporter subunit EIIC “AB1A69_09025”
*ccpA*-Entfm-com	Carbon catabolite control protein A	Catabolite control protein A “AB1A69_01735”
*empA*-Entfm-hosp	*ebpAfm* (fms1)/Endocarditis and biofilm-associated pili A	SpaA isopeptide-forming pilin-related protein “AB1A69_04555”
*empB*-Entfm-hosp	*ebpBfm* (fms5)/Endocarditis and biofilm-associated pili B	SpaH/EbpB family LPXTG-anchored major pilin “AB1A69_00310”
*empC*-Entfm-hosp	*ebpCfm* (fm9)/Endocarditis and biofilm-associated pili C	SpaH/EbpB family LPXTG-anchored major pilin “AB1A69_04565”
*fms11*-Entfm-hosp	*E. faecium* surface protein 11	SpaH/EbpB family LPXTG-anchored major pilin
*fms13*-Entfm	*E. faecium* surface protein 13	SpaH/EbpB family LPXTG-anchored major pilin
*fms14*-Entfm-hosp	*E. faecium* surface protein 14	LPXTG family cell surface protein Fms14
*fms15*-Entfm-hosp	*E. faecium* surface protein 15	LPXTG cell wall anchor domain-containing protein
*fms16*-Entfm-hosp	*E. faecium* surface protein 16	SpaH/EbpB family LPXTG-anchored major pilin
*fms17*-Entfm-com	*E. faecium* surface protein 17	LPXTG cell wall anchor domain-containing protein
*fms19*-Entfm-hosp	*E. faecium* surface protein 19	SpaH/EbpB family LPXTG-anchored major pilin
*fnm*-Entfm-com	Fibronectin-binding protein of *E. faecium*	Fibronectin-binding protein EfbA
*gls20*-Entfm	General stress protein GlsB20	Asp23/Gls24 family envelope stress response protein
*gls33*-Entfm	General stress protein Gls33	Asp23/Gls24 family envelope stress response protein
*glsB*-Entfm	General stress protein GlsB (gls33-glsB cluster)	GlsB/YeaQ/YmgE family stress response membrane protein
*glsB1*-Entfm	General stress protein GlsB1	GlsB/YeaQ/YmgE family stress response membrane protein
*sagA*-Entfm-hosp	Secreted antigen A	Hypothetical protein
*scm*-Entfm-hosp	(*fms10* (MSCRAMM)/Second collagen adhesin of *E. faecium*	Collagen-binding MSCRAMM adhesin “AB1A69_11605”
*sgrA*-Entfm-hosp	Serine-glutamate repeat containing protein A	LPXTG-anchored fibrinogen/nidogen-binding adhesin SgrA “AB1A69_03970”

## Data Availability

The original contributions presented in this study are included in the article/supplementary material. Further inquiries can be directed to the corresponding author.

## Supplementary Materials

The following supporting information can be downloaded at: https://www.mdpi.com/article/10.3390/microorganisms14010035/s1, Figure S1: Circular genome mapping of *E. faecium* strain 1702 of size 2,621,416. Genome visualization consists of five rings. The outer concentric circle followed by the second circle represents the position of protein-coding genes and RNAs on forward and reverse strands, respectively. Locus tags and/or gene names are displayed in blue color. The middle circle represents draft genome scaffolds colored in gray followed by a ring showing GC content (black). Next, the inner ring shows GC Skew [(G–C)/(G+C)] distribution. Positive GC Skew is indicated in red and negative GC skew in sky blue; Table S1: Primers used for *Enterococcus* spp. isolates’ identification; Table S2: Phenotypic traits of 53 LABs isolated from bottarga; Table S3: Enterocin A cluster (region size: 20126 bp; region name: NODE_5_length_193743_cov_51698124.9.AOI_01); Table S4: Enterocin B cluster (region size: 21494; region name: NODE_4_length_269662_cov_50586566.0.AOI_01); Table S5: List of probiotic marker genes identified in *E. faecium* strain 1702.
