# Supplementary material for: Deciphering the Genomic Traits of Multi-Enterocin-Producing E. faecium 1702 from Bottarga: A WGS-Based Characterization
Source: Microorganisms. 2025 Dec 23;14(1):35. doi: 10.3390/microorganisms14010035 (PMC12844089; doi:10.3390/microorganisms14010035)
Supplement: Supplementary file 1 [file microorganisms-14-00035-s001.zip › microorganisms-3936474-supplementary.pdf]

Supplementary files

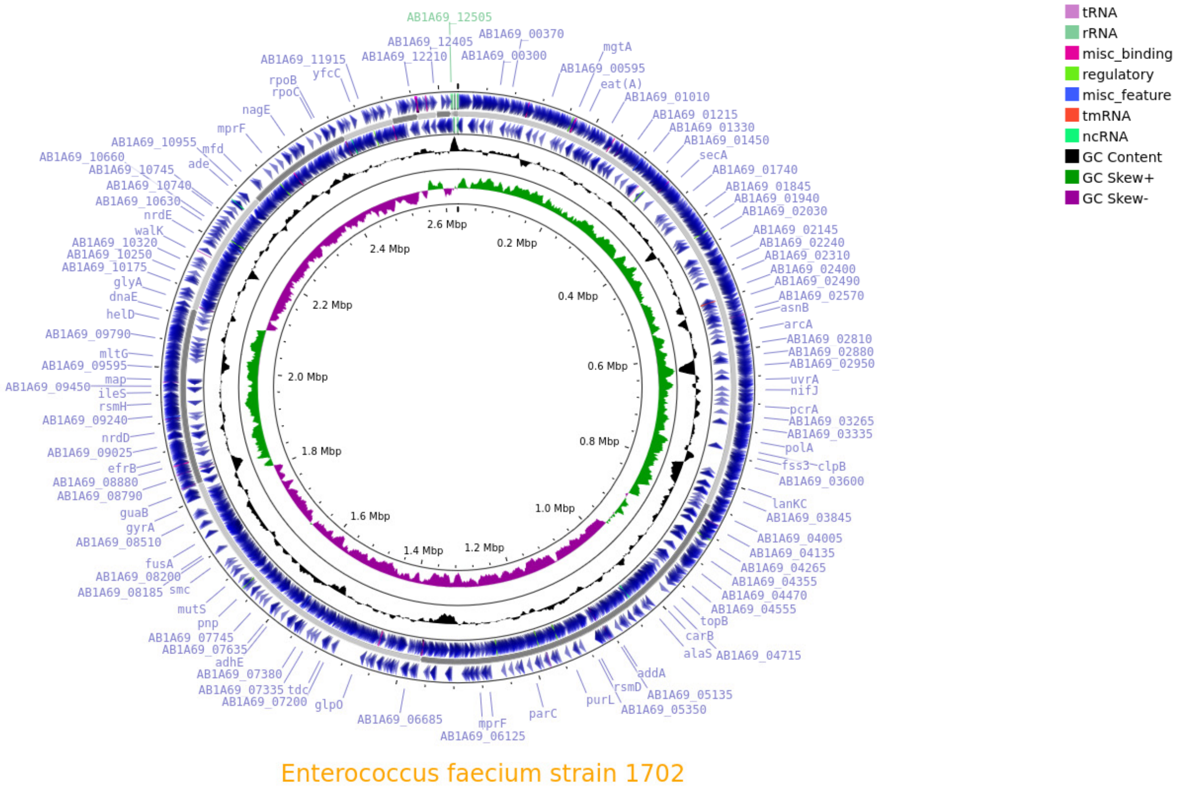

Figure S1. Circular genome mapping of *E. faecium* strain 1702 of size 2,621,416. Genome visualization consists of five rings. The outer concentric circle followed by the second circle represents the position of protein-coding genes and RNAs on forward and reverse strands, respectively. Locus tags and/or gene names are displayed in blue color. The middle circle represents draft genome scaffolds colored in gray followed by a ring showing GC content (black). Next, the inner ring shows GC Skew [(G–C)/(G+C)] distribution. Positive GC Skew is indicated in red and negative GC skew in sky blue.

Table S1. Used primers for *Enterococcus* spp isolates identification

**Amplified genes**	**Primer sequence (5′ - 3′)**	**Amplification conditions**	**Size of PCR products (bp)**	**References**
tuf (Enterococcus)	F-TACTGACAAACCATTCATGATG<br>R- AACTTCGTCACCAACGCGAAC	95°C/5; 35 cycles: 95°C/30s, 55°C/30s, and 1 min at 72°C/1min; 72°C/7 min	112	Ke et al. 1999
ddl$_{E. faecalis}$	F-ATCAAGTACAGTTAGTCT<br>R-ACGATTCAAAGCTAACTG	94°C/5mIn; 30 cycles: 94°C/1mIn, 54°C/1mIn,72°C/1mIn; 72°C/10min	941	Dutka-Malen et al., 1995
ddl$_{E. faecium}$	F-TAGAGACATTGAATATGCC<br>R-TCGAATGTGCTACAATC		550	
vanC-1 (E. gallinarum)	F-GGTATCAAGGAAACCTC<br>R- CTTCCGCCATCATAGCT		822	
vanC-2,vanC-3 (E. casseliflavus/E. flavescence)	F-CTCCTACGATTCTCTTG<br>R- CGAGCAAGACCTTTAAG		439	

Table S2. Phenotypic traits of 53 LAB isolated from bottarga.

Isolaes	origins	Identification	Eneterocin	Inhibition spectrum											Activities				
				L.monocytogenes	*S. aureus*	*E. feacalis*	*E. faecium*	*V.alginolyticus*	*V. anguillarum*	*E. coli*	*K.pneumiae*	*A. baumannii*	*P. aeruginosa*	*S. typhimirum*	B. cereus	DNase	Gelatinase	Caseinase	Lipase
P1	T2	Efc	*entB*	+	-	+	-	-	-	-	-	-	-	-	-	-	-	-	-
P2	T2	ND	-	-	-	-	-	-	-	-	-	-	-	-	-	-	-	-	-
P3	T2	ND	-	-	-	-	-	-	-	-	-	-	-	-	-	-	-	-	-
P4	T1	ND	-	-	-	-	-	-	-	-	-	-	-	-	-	-	-	-	-
P5	T1	Efm	-	++	-	-	++	-	-	-	-	-	-	-	-	-	-	-	-
P6	T2	Efm	-	-	++	-	-	-	-	++	-	++	++	-	-	-	-	+	-
**P7 (Efm1702)**	**T1**	**Efm**	***entA, entB, entX***	**++**	**-**	**-**	**++**	**-**	**-**	**-**	**-**	**-**	**-**	**-**	**-**	**-**	**-**	**-**	**-**
P8	T2	Efc	-	-	-	-	-	-	-	++	-	-	-	-	-	-	-	-	-
P9	T2	ND	-	-	-	-	-	-	-	-	-	-	-	-	-	-	-	-	-
P10	T2	Efc	-	-	-	-	++	-	-	-	-	-	-	-	-	-	-	-	-
P11	T2	Efc	*entB*	++	-	-	++	-	-	-	-	-	-		-	-	+	-	+
P12	T2	Efc	-	-	-	-	-	-	++	-	-	-	-	-	-	-	-	-	-
P13	T1	ND	-	-	-	-	-	-	-	-	-	-	-	-	-	-	-	-	-
P14	T2	ND	-	-	-	-	-	-	-	-	-	-	-	-	-	-	-	-	+
P15	T2	Efc	*entB*	++	-	-	-	-	-	-	-	-	-	-	-	-	-	-	-
P16	T2	Efc	*entB*	++	-	-	-	-	-	-	-	-	-	-	-	-	-	-	+
P17	T2	Efm	*entB*	-	-	-	-	-	-	-	-	++	-	-	-	-	-	-	-
P18	T1	Efm	-	-	-	-	-	++	++	-	-	-	-	-	-	-		+	-
P19	M	Efm	*entB*	+++	-	-	+++	-	-	-	-	-	-	-	-	-	-	-	-
P20	M	ND	-	-	-	-	-	-	-	-	-	-	-	-	-	-	+	-	-
P21	M	ND	-	-	-	-	-	-	-	-	-	-	-	-	-	-	-	-	+
P22	M	Efm	*ent*1071	-	++	-	-	-	-	-	-	-	-	-	-	-	-	-	+

			A/B																
P23	M	ND	-	-	-	-	-	-	-	-	-	-	-	-	-	-	-	-	-
P24	M	ND	-	-	-	-	-	-	-	-	-	-	-	-	-	-	-	-	-
P25	M	ND	-	-	-	-	-	-	-	-	-	-	-	-	-	-	+	-	+
P26	M	Efm	-	-	-	-	-	++	++	-	-	-	-	-	-	-	+	+	-
P27	M	ND	-	-	-	-	-	-	-	-	-	-	-	-	-	-	+	-	-
P28	M	Lla	*ent1071 A*/B	-	-	-	-	-	-	-	-	++	-	-	+	-	+	-	-
P29	M	ND	-	-	-	-	-	-	-	-	-	-	-	-	-	-	-	-	+
P30	M	ND	-	-	-	-	-	-	-	-	-	-	-	-	-	-	-	-	-
P31	M	ND	-	-	-	-	-	-	-	-	-	-	-	-	-	-	+	-	-
P32	M	Lla	*ent1071 A*/B	-	-	-	-	++	++	-	-	-	-	-	-	-	-	-	-
P33	M	Efm	-	-	-	-	-	-	-	++	-	-	-	-	-	-	-	+	-
P34	M	Efm	-	-	-	-	-	-	++	-	-	-	-	++	-	-	-	+	-
P35	M	ND	-	-	-	-	-	-	-	-	-	-	-	-	-	-	-	-	-
P36	M	Efc	-	-	-	-	-	-	++	-	-	-	++	-	-	+	+	+	-
P37	M	ND	-	-	-	-	-	-	-	-	-	-	-	-	-	-	-	-	-
P38	M	Efc	-	-	-	-	-	-	++	++	-	++	-	++	-	-	-	-	-
P39	M	ND	-	-	-	-	-	-	-	-	-	-	-	-	-	-	-	-	-
P40	M	Efc	-	-	-	-	-	-	++	-	-	-	-	-	-	-	+	+	+
P41	M	Efc	*ent1071 A*/B	-	-	-	-	-	-	-	-	-	-	++	-	-	-	-	+
P42	M	Efc	*ent1071 A*/B + *entB*	++	-	++	-	-	-	-	-	-	-	-	-	-	+	-	-
P43	M	Efc	*ent1071 A*/B	++	-	++	-	-	-	++	-	++	-	-	-	-	+	-	-
P44	M	Efc	*ent1071 A*/B	-	-	++	-	-	-	-	-	-	-	-	+	-	+	+	-
P45	M	Efc	-	-	-	++	-	-	-	-	-	-	-	-	-	-	-	+	+
P46	M	Efc	-	-	-	-	++	-	-	-	-	-	-	-	-	-	-	+	-
P47	M	Ec	-	-	-	-	++	-	++	-	-	-	-	-	-	-	+	-	-
P48	M	ND	-	-	-	-	-	-	-	-	-	-	-	-	-	-	-	-	-
P49	M	ND	-	-	-	-	-	-	-	-	-	-	-	-	-	-	-	-	-
P50	M	Ec	-	-	-	++	-	-	-	-	-	-	-	-	-	-	+	+	-
P51	M	Ec	-	-	-	++	-	-	-	-	-	-	-	-	-	-	+	-	-
P52	M	ND	-	-	-	-	-	-	-	-	-	-	-	-	-	-	-	-	+
P53	M	Lla	-	-	-	++	-	-	-	-	+	-	-	-	-	-	-	-	-

E.fc: *E. faecalis*, Efm : *E. faecium*, Ec : *E. casseluflavis*, Lla : *L. lactis*, ND. Not determined. T1 and T2: Red tuna bottarga. M: Wild mullet bottarga.

Table S3. Enterocin A cluster (region size: 20126 bp; region name: NODE_5_length_193743_cov_51698124.9.AOI_01)

Orf	Gene_name	Gene_start	Gene_end	Gene_strand	Function
orf00001	orf00001	16	144	-	
orf00003	orf00003	402	788	-	
orf00004	orf00004	785	1306	-	Uncharacterized membrane protein YteJ OS=*Bacillus subtilis* (strain 168) OX=224308 GN=yteJ PE=4 SV=1
orf00005	orf00005	1308	2342	-	Putative signal peptide peptidase SppA OS=*Enterococcus faecium* OX=1352 GN=sppA PE=3 SV=1
orf00006	orf00006	2375	3673	-	Transport protein ComB OS=*Streptococcus pneumoniae* (strain ATCC BAA-255 / R6) OX=171101 GN=comB PE=3 SV=1
orf00008	LanT	3759	5912	-	Lactococcin-G-processing and transport ATP-binding protein lagD
orf00010	orf00010	6170	6406	+	Bacteriocin_IIc
orf00011	Lactacin_F subunit_lafX	6425	6640	+	Bacteriocin_IIc; Mersacidin; 113.2;Lactacin_F,subunit_lafX
orf00013	orf00013	6696	6824	+	
orf00014	orf00014	7036	7164	-	
orf00016	orf00016	7344	8096	-	putative piscicolin 126 response regulator
orf00017	orf00017	8111	9358	-	Bacteriocin production related histidine kinase
orf00018	orf00018	9434	9580	-	Lactococcin
orf00019	EntA_Immun	9684	9995	-	AF240561_2 putative immunity protein EntI
orf00020	Enterocin_A	9997	10194	-	bacteriocinII; Bacteriocin_II; 81.2;Enterocin_A
orf00026	orf00026	10588	12315	-	Glycine betaine/carnitine transport permease protein GbuB OS=*Listeria monocytogenes* serotype 1/2a (strain 10403S) OX=393133 GN=gbuB PE=1 SV=1
orf00028	ABC	12308	13435	-	Glycine betaine transport ATP-binding protein OpuAA OS=*Lactococcus lactis* subsp. *lactis* (strain IL1403) OX=272623 GN=opuAA PE=3 SV=1
orf00030	orf00030	13478	13642	+	
orf00032	orf00032	13688	14332	-	Exu regulon transcriptional regulator OS=*Shigella flexneri* OX=623 GN=exuR PE=3 SV=1
orf00035	orf00035	14426	14560	-	
orf00037	orf00037	14562	16694	-	Fe(2+) transporter FeoB OS=*Staphylococcus aureus* (strain MW2) OX=196620 GN=feoB PE=3 SV=1
orf00039	orf00039	16691	17206	-	Fe(2+) transport protein A/Fe(2+) transporter FeoB fusion protein OS=*Porphyromonas gingivalis* (strain ATCC BAA-308 / W83) OX=242619 GN=feoB1 PE=3 SV=1
orf00040	orf00040	17309	17566	-	
orf00041	orf00041	17610	17789	+	Glutaredoxin-like protein NrdH OS=Lactococcus lactis subsp. lactis (strain

					IL1403) OX=272623 GN=nrdH PE=3 SV=2
orf00045	orf00045	17948	20107	+	Ribonucleoside-diphosphate reductase subunit alpha 2 OS=*Mycolicibacterium smegmatis* (strain ATCC 700084 / mc(2)155) OX=246196 GN=nrdE2 PE=1 SV=1
sORF_1	sORF_1	6022	6141	+	
sORF_2	sORF_2	7176	7292	+	
sORF_3	sORF_3	10383	10568	-	
sORF_4	sORF_4	9592	9684	-	
sORF_5	sORF_5	7161	7289	-	
sORF_6	sORF_6	5943	6041	-	
sORF_7	sORF_7	3658	3771	-	

Table S4. Enterocin B cluster (region size: 21494; region name: NODE_4_length_269662_cov_50586566.0.AOI_01)

Orf	Gene_name	Gene_start	Gene_end	Gene_strand	Function
orf00001	orf00001	203	814	+	Putrescine carbamoyltransferase OS=Pediococcus pentosaceus (strain ATCC 25745 / CCUG 21536 / LMG 10740 / 183-1w) OX=278197 GN=ptcA PE=3 SV=1
orf00003	orf00003	926	2314	+	Probable agmatine/putrescine antiporter AguD OS=Lactococcus lactis subsp. lactis (strain IL1403) OX=272623 GN=aguD PE=3 SV=1
orf00005	orf00005	2343	3407	+	Putative agmatine deiminase OS=*Lactobacillus sakei* subsp. *sakei* (strain 23K) OX=314315 GN=aguA PE=3 SV=1
orf00006	orf00006	3404	4393	+	Carbamate kinase 1 OS=Enterococcus faecalis (strain ATCC 700802 / V583) OX=226185 GN=arcC1 PE=1 SV=2
orf00007	orf00007	4511	5638	+	Putative agmatine deiminase 1 OS=Listeria monocytogenes serotype 4b (strain F2365) OX=265669 GN=aguA1 PE=3 SV=1
orf00010	orf00010	5656	6453	+	HTH-type transcriptional regulator GlvR OS=Bacillus subtilis (strain 168) OX=224308 GN=glvR PE=2 SV=1
orf00017	orf00017	9096	9290	+	
orf00018	orf00018	9459	9581	+	ggmotif
orf00019	orf00019	9581	9778	+	
orf00021	orf00021	9794	9949	-	
orf00022	Enterocin_B	9998	10213	-	ggmotif; ComC; L_biotic_typeA; Antimicrobial17; Bacteriocin_IIc; 82.2;Enterocin_B
orf00026	orf00026	10798	11019	-	
orf00027	Enterocin_X_chain_beta	10798	11019	-	Bacteriocin_IIc; 560.1;rSAM-modified_RiPP_06197.2;Enterocin_X_chain_beta
orf00028	Enterocin_X_chain_alpha	11134	11301	-	leaderLanM; LE-MER+2PEP; L_biotic_typeA; Bacteriocin_IIc; 96.2;Enterocin_X_chain_alpha
orf00030	orf00030	11782	11988	+	DNA repair protein homolog YozK OS=*Bacillus subtilis* (strain 168) OX=224308 GN=yozK PE=5 SV=1
orf00032	orf00032	12102	12317	+	
orf00034	orf00034	12811	13656	+	Transcription antiterminator LicT OS=*Bacillus subtilis* (strain 168) OX=224308 GN=licT PE=1 SV=1

orf00036	orf00036	13649	15505	+	PTS system beta-glucoside-specific EIIBCA component OS=Bacillus subtilis (strain 168) OX=224308 GN=bglP PE=3 SV=2
orf00037	orf00037	15517	16944	+	6-phospho-beta-glucosidase BglA OS=*Escherichia coli* (strain K12) OX=83333 GN=bglA PE=1 SV=2
orf00038	orf00038	17212	17631	+	
orf00040	orf00040	17746	18375	-	Putative 2-succinyl-6-hydroxy-2
orf00042	orf00042	18572	18742	-	
orf00047	orf00047	19006	19179	-	
orf00050	orf00050	19465	20793	+	Aminopeptidase C OS=*Streptococcus thermophilus* OX=1308 GN=pepC PE=3 SV=1
sORF_1	sORF_1	6450	6689	+	
sORF_10	sORF_10	8317	8424	+	
sORF_11	sORF_11	8611	8721	+	
sORF_12	sORF_12	8970	9068	+	
sORF_13	LanT	10391	10504	+	Lactococcin-G-processing and transport ATP-binding protein lagD
sORF_14	sORF_14	10592	10771	+	
sORF_15	sORF_15	12292	12462	+	Putative UV-damage repair protein UvrX OS=*Bacillus subtilis* (strain 168) OX=224308 GN=uvrX PE=3 SV=2
sORF_16	sORF_16	18823	18972	+	
sORF_17	sORF_17	18857	19009	-	
sORF_18	sORF_18	18760	18906	-	
sORF_19	sORF_19	18443	18550	-	
sORF_2	sORF_2	6579	6689	+	
sORF_20	sORF_20	16997	17116	-	
sORF_21	sORF_21	12389	12484	-	
sORF_22	sORF_22	10661	10801	-	
sORF_23	sORF_23	10582	10677	-	
sORF_24	sORF_24	10437	10556	-	
sORF_25	sORF_25	9371	9460	-	
sORF_26	sORF_26	8887	8991	-	
sORF_27	sORF_27	8290	8391	-	
sORF_28	sORF_28	7799	8095	-	
sORF_29	sORF_29	7799	8002	-	
sORF_3	sORF_3	6690	6794	+	
sORF_30	sORF_30	7374	7466	-	
sORF_31	sORF_31	6991	7095	-	
sORF_32	sORF_32	6798	6890	-	
sORF_33	sORF_33	6634	6768	-	
sORF_4	sORF_4	6998	7171	+	
sORF_5	sORF_5	7298	7519	+	
sORF_6	sORF_6	7376	7519	+	

sORF_7	sORF_7	7539	7658	+
sORF_8	sORF_8	7962	8081	+
sORF_9	sORF_9	8068	8166	+

Table S5. List of probiotic marker genes identified in *E. faecium* strain 1702.

**Gene**	**Function/product**
usp	Universal stress proteins (broad spectrum of cellular responses to biotic and abiotic stressors, ion scavenging, hypoxia responses, motility of cell, and regulation of growth and development of cells)
**Adaptability and stress tolerance response related genes of gastrointestinal environment**	
**Oxidative stress**	
spx (suppressor of ClpP and ClpX phenotypes)	Global transcription regulator Spx
npr (EC:1.11.1.1)	NADH peroxidase
ytpP	Thioredoxin
trxA (ko:K03671)	Thioredoxin
nox (EC: 1.6.3.4)	NADH oxidase
trxB (EC:1.8.1.9)	Thioredoxin reductase (**NADP-thioredoxin reductase)**
nqo1 (ko:K19784)	NADPH-dependent FMN reductase (FMN reductase [NAD(P)H])
pyrK (ko:K02823)	Dihydroorotate oxidase (Responsible for channeling the electrons from the oxidation of dihydroorotate from the FMN redox center in the PyrD type B subunit to the ultimate electron acceptor NAD( )
pyrD (EC :1.3.5.2, EC :1.3.98.1)	dihydroorotate dehydrogenase (quinone)) ; Catalyzes the conversion of dihydroorotate to orotate
mntC (ko:K19976)	Manganese transport system permease protein
mntA (ko:K19975,ko:K19976)	Manganese transport system ATP-binding protein [EC:7.2.2.5]
mntB (EC:3.6.3.35)	Manganese transport system permease protein
msrA (EC: 1.8.4.11)	Peptide-methionine (S)-S-oxide reductase. Has an important function as a repair enzyme for proteins that have been inactivated by oxidation. Catalyzes the reversible oxidation-reduction of methionine sulfoxide in proteins to methionine
msrB (EC:1.8.4.12)	Peptide-methionine (R)-S-oxide reductase (peptide methionine sulfoxide reductase)
nrdH	Glutaredoxin (ko:K06191)
sodA	Superoxide dismutase (EC1.15.1.1)
**Heat-shock stress**	
HSP20	HSP20 family protein (ko:K13993)
grpE	heat shock protein GrpE
groES	heat-shock chaperonin proteins 10 (Hsp10 or cpn10)
groEL	heat-shock chaperonin protein 60 (Hsp60 or Cpn60)
hrcA	Heat-inducible transcription repressor HrcA ; Negative regulator of class I heat shock genes (grpE- dnaK-dnaJ and groELS operons). Prevents heat-shock induction of these operons
hslO	33 kDa chaperonin

dnaK	Chaperone protein DnaK
dnaJ	Chaperone protein DnaJ
ctsR	Transcriptional regulator of stress and heat shock response CtsR
hslV	ATP-dependent HslUV protease, peptidase subunit HslV (EC:3.4.25.2)
hslU	ATP-dependent HslUV protease ATP-binding subunit HslU (ko:K03667)
**Cold-shock stress**	
cspA (ko:K03704)	cold shock protein
cspB (ko:K03704)	cold shock protein
cspC (ko:K03704	cold shock protein
*csp*D (ko:K03704)	cold shock protein
ctsR	Transcriptional regulator of stress and heat shock response CtsR
clp operon (*clpX, clpB, clpP, clpC, clpE*)	ATP-dependent Clp protease
lepA	Ribosomal elongation factor 4 (EF4), promotes survival or death, depending on the severity of stress.
**Acid stress**	
atpA (EC:3.6.3.14)	ATP synthase subunit alpha; Produces ATP from ADP in the presence of a proton gradient across the membrane. The alpha chain is a regulatory subunit)
atpB (ko:K02108)	ATP synthase subunit a, it plays a direct role in the translocation of protons across the membrane
atpC (ko:K02114)	ATP synthase epsilon chain
atpF (ko:K02109)	ATP synthase subunit b; Component of the F(0) channel, it forms part of the peripheral stalk, linking F(1) to F(0)
atpE (ko:K02110)	ATP synthase subunit c; F(1)F(0) ATP synthase produces ATP from ADP in the presence of a proton or sodium gradient.
atpD (EC :3.6.3.14)	ATP synthase subunit beta
atpH (ko:K02113)	ATP synthase subunit delta; F(1)F(0) ATP synthase produces ATP from ADP in the presence of a proton or sodium gradient.
atpG (ko:K02115)	ATP synthase gamma chain; The gamma chain is believed to be important in regulating ATPase activity and the flow of protons through the CF(0) complex
nhaK	Sodium/hydrogen exchanger (sodium-proton (Na+/H+) antiporters)
nhaC	Sodium/hydrogen exchanger (sodium-proton (Na+/H+) antiporters)
nhaP1	Sodium/hydrogen exchanger (sodium-proton (Na+/H+) antiporters)
plsC	1-acyl-sn-glycerol-3-phosphate acyltransferase [EC:2.3.1.51]
pyK	Pyruvate kinase [EC:2.7.1.40]
**Bile tolerence**	
ppaC	Manganese-dependent inorganic pyrophosphatase, maintain surface tension and keep membrane integrity

arsB	Sodium Bile acid symporter
cbh (EC :3.5.1.24)	Choloylglycine hydrolase
cfa (EC:2.1.1.79)	cyclopropane-fatty-acyl-phospholipid synthase (Mycolic acid cyclopropane synthetase, bile salt hydrolase). It enhance lipid synthesis. The cyclopropane fatty acid defends probiotics from some adverse environments, such as exposure to acid, bile salt or other pollutants.
glnA (EC: 6.3.1.2)	glutamine synthetase
mleS (EC:1.1.1.38,4.1.1.101)	Malic enzyme, NAD binding domain
murE (EC:6.3.2.13,6.3.2.7)	malolactic enzyme (MLE) **(**UDP-N-acetylmuramoyl-L-alanyl-D-glutamyl-L-lysine synthetase)
oppA (Ko:K15580)	Oligopeptide transport system substrate-binding protein
oppB (ko:K15581)	Oligopeptide transport system permease protein
oppC (Ko :ko:K15582)	Oligopeptide transport system permease protein
oppD (Ko :ko:K02031,ko:K15583)	Oligopeptide transport system ATP-binding protein
oppF (Ko :ko:K10823)	Oligopeptide transport system ATP-binding protein
**Alkalin stress**	
aspS (EC: 6.1.1.12)	Aspartate–tRNA ligase
asp1	Asp23 family, cell envelope-related function (Accessory Sec system protein Asp1)
yloU	Asp23 family, cell envelope-related function, putative alkaline-shock protein
nhaK + *nhaC* +*nhaP1*	Sodium/hydrogen exchanger (sodium-proton (Na+/H+) antiporters)
WQ51_04310	Alkaline shock protein (Asp23) family (ko:K10947)
**Osmotic shock tolerance**	
opuAA (EC:3.6.3.32; ko:K02000)	Glycine betaine transport ATP-binding protein OpuAA
opuCA (ko:K05847)	Glycine betaine/carnitine/choline transport ATP binding protein OpuCA
opuCB (ko:K05846)	Binding-protein-dependent transport system inner membrane component
opuCC (ko:K05845)	Substrate binding domain of ABC-type glycine betaine transport system
opuCD (ko:K05846)	Binding-protein-dependent transport system inner membrane component
proWX (ko:K05845,ko:K05846)	Glycine betaine/proline transport system permease protein
**Alcohol resistance**	
gap (EC:1.2.1.12)	Glyceraldehyde-3-phosphate dehydrogenase
**Vitamin biosynthesis**	
ribU	Mediates riboflavin uptake, may also transport flavin mononucleotide (FMN) and roseoflavin.
ribH (rib operon)	Catalyzes the formation of 6,7-dimethyl-8-ribityllumazine by condensation of 5-amino-6-(D-ribitylamino)uracil with 3,4-dihydroxy-2-butanone 4-phosphate. This is the penultimate step in the biosynthesis of riboflavin
ribBA (rib operon)	Catalyzes the conversion of D-ribulose 5-phosphate to formate and 3,4-dihydroxy-2-butanone 4-phosphate

ribF	Riboflavin kinase and FMN adenylyltransferase
ribE	Riboflavin synthase
ribD	Converts 2,5-diamino-6-(ribosylamino)-4(3h)-pyrimidinone 5'-phosphate into 5-amino-6-(ribosylamino)-2,4(1h,3h)- pyrimidinedione 5'-phosphate
**Immunomodulation**	
dltA (EC:6.1.1.13)	D-alanine--poly(phosphoribitol) ligase subunit 1
dltB (ko:K03739)	Membrane protein involved in D-alanine export
dltC (EC:6.1.1.13)	D-alanine--poly(phosphoribitol) ligase subunit 2
dltD (ko:K03740)	D-alanine transfer protein
rpoA	α subunit of RNA polymerase
rpoB (EC 2.7.7.6)	β subunit of RNA polymerase
fusA (ko:K02355)	Translation elongation factor
pyrG (EC 6.3.4.2)	CTP synthetase
**Bacteriocin production**	
entA	Antibacterial activity
enB	Antibacterial activity
entX	Antibacterial activity
**Production of lactic acid**	
dld (EC:1.1.5.12)	D-lactate dehydrogenase
**Cellular adhesion and gut persistence**	
mapA (EC:2.4.1.8 ; ko:K00691)	Maltose phosphorylase
pgi (EC: 5.3.1.9 ; ko:K01810)	Glucose-6-phosphate isomerase
fbpA (DUF814, FbpA)	fibronectin-binding protein A
*lspA (*EC:3.4.23.36)	lipoprotein signal peptidase II
Tuf (ko:K02358)	elongation factor Tu
srtA (EC:3.4.22.70)	sortase A
eno (EC:4.2.1.11)	Enolase, catalyzes the reversible conversion of 2-phosphoglycerate into phosphoenolpyruvate. It is essential for the degradation of carbohydrates via glycolysis
mapA (EC: 2.4.1.8)	maltose phosphorylase
celD (ko:K02761)	The phosphoenolpyruvate-dependent sugar phosphotransferase system (PTS), a major carbohydrate active - transport system, catalyzes the phosphorylation of incoming sugar substrates concomitant with their translocation across the cell membrane
celC (EC:2.7.1.196, 2.7.1.205 ; ko:K02759)	PTS system, Lactose/Cellobiose specific IIA subunit
celA(EC:2.7.1.196,2.7.1.205 ;ko:K02760)	Cellobiose PTS system EIIB component
celR (ko:K03491)	PTS system, EIIA 2
celB(EC: 2.7.1.196,2.7.1.205 ; ko:K02760)	PTS system, Lactose/Cellobiose specific IIB subunit
**Anticancer genes**	
ansB (EC:3.5.1.1)	L-asparaginase
glsA (EC:3.5.1.2)	L-glutaminase
arcA (EC:3.5.3.6)	Arginine deiminase
